# Phase-Field Model for the Simulation of Brittle-Anisotropic and Ductile Crack Propagation in Composite Materials

**DOI:** 10.3390/ma14174956

**Published:** 2021-08-30

**Authors:** Christoph Herrmann, Daniel Schneider, Ephraim Schoof, Felix Schwab, Britta Nestler

**Affiliations:** 1 Institute of Applied Materials (IAM-CMS), Karlsruhe Institute of Technology (KIT), Kaiserstrasse 12, D-76131 Karlsruhe, Germany; daniel.schneider@kit.edu (D.S.); ephraim.schoof@kit.edu (E.S.); britta.nestler@kit.edu (B.N.); 2 Institute of Digital Materials Science (IDM), Karlsruhe University of Applied Sciences, Moltkestrasse 30, D-76133 Karlsruhe, Germany; 3 Groupe de Physique des Matériaux, University of Rouen, CNRS, Avenue de l’Université, F-76800 Saint Etienne du Rouvray, France; felix.schwab@univ-rouen.fr

**Keywords:** phase-field, multiphase-field, grey cast iron, brittle fracture, ductile fracture, anisotropic fracture

## Abstract

In this work, a small-strain phase-field model is presented, which is able to predict crack propagation in systems with anisotropic brittle and ductile constituents. To model the anisotropic brittle crack propagation, an anisotropic critical energy release rate is used. The brittle constituents behave linear-elastically in a transversely isotropic manner. Ductile crack growth is realised by a special crack degradation function, depending on the accumulated plastic strain, which is calculated by following the $J_{2}$-plasticity theory. The mechanical jump conditions are applied in solid-solid phase transition regions. The influence of the relevant model parameters on a crack propagating through a planar brittle-ductile interface, and furthermore a crack developing in a domain with a single anisotropic brittle ellipsoid, embedded in a ductile matrix, is investigated. We demonstrate that important properties concerning the mechanical behaviour of grey cast iron, such as the favoured growth of cracks along the graphite lamellae and the tension–compression load asymmetry of the stress–strain response, are covered by the model. The behaviour is analysed on the basis of a simulation domain consisting of three differently oriented elliptical inclusions, embedded in a ductile matrix, which is subjected to tensile and compressive load. The material parameters used correspond to graphite lamellae and pearlite.

## 1. Introduction

For the numerical prediction or analysis of fractures and the associated failure of components, the investigation of the formation and propagation of cracks is necessary. In the study of crack development, some physical models and numerical methods have gained acceptance over time. Important approaches of the “old guard”, for example, are discrete formulations, with cohesion zone models [1] as the most prominent representative, and continuum damage models [2,3]. With these approaches, it is, however, difficult to capture phenomena such as crack branching and merging as well as crack nucleation processes in arbitrarily complex systems. These phenomena can be intrinsically captured when using a phase-field approach to crack modelling, which is why such methods are enjoying growing popularity [4,5,6]. The evolution of cracks is described within the phase-field framework by a regularised representation of the Griffith criterion [7], where the degree of damage is given by a scalar continuous phase field. Subsequently, a phase-field model is then presented that allows the simulation of crack development processes in composite materials, consisting of brittle-anisotropic and ductile components. This is done by analogy with lamellar graphite cast iron, in which the matrix of the cast iron behaves in a ductile manner and the inclusions in a brittle-anisotropic manner. Subsequently, the constituents of cast iron with lamellar graphite, the pearlitic matrix, and the graphite particles are represented in an idealised form. The ductile phase is considered to be purely pearlitic and is modelled as a homogeneous ductile isotropic phase, according to the $J_{2}$-plasticity theory with linear hardening. The effects of the microscopic structure of pearlite, consisting of ferrite and cementite, are only considered in a homogenised manner. Brittle phases are related to lamellar graphite. As inclusions, they are similar to graphite lamellae in terms of shape and mechanical properties. However, they are larger. On the atomistic level, lamellar graphite is made up of interconnected graphene layers. Within the graphene layers, covalent bonds exist between the carbon atoms. The different graphene layers are connected by weak van der Waals forces [8,9,10]. Due to its atomistic structure, lamellar graphite possesses a transverse isotropy, with regard to its elastic stiffness and critical energy release rate, and so do the anisotropic brittle phases. For multiphase materials, the proposed model is the first phase-field crack model we are aware of, which combines brittle-anisotropic and ductile crack propagation. For this purpose, two existing phase-field models for crack development are combined and introduced into the multiphase context: one for brittle-anisotropic crack development and one for ductile crack development.

The formulation of the previous publication by Prajapati et al. [11] serves as a model for the description of brittle-anisotropic crack development. Here, the critical energy release rate is directionally dependent. Alternative approaches for anisotropic crack growth are based on multiplying the divergence term of the evolution equation of the crack phase field with an anisotropy tensor, resulting in a directional dependency (see, e.g., [12,13,14]). In the context of gradient energy, however, the physical representation of such an anisotropy tensor is still unclear [14].

When choosing a possible ductile crack model, various approaches are available, which are briefly summarised below. The model of Duda et al. [15] was one of the first models to integrate plasticity into a phase-field crack model, so as to simulate brittle fractures in plastic materials. Some important phenomenological characteristics of ductile fracture, reported in experimental literature, could be reproduced in Ambati et al. [16] by coupling the degradation function with the plastic strain state. In Ambati et al. [17], the model was extended to finite strains. In the context of thermoplasticity, a thermodynamically consistent phase-field crack model for brittle to ductile fractures is introduced in Miehe et al. [18] at large deformations. As an extension to this, they also worked on porous-isotropic plasticity in Miehe et al. [19]. In Kuhn et al. [20], an elastoplastic phase-field fracture model, where a monolithic solution is possible, is proposed. In Miehe et al. [21], gradient plasticity is used at finite strains in order to model ductile fracture in a variational-based phase-field framework. In a recently published book article Alessi et al. [6], prominent phase-field models for ductile crack growth are compared, and a study of their predictive capabilities is conducted.

Based on the investigations of the anisotropic brittle crack model of our previously published work, Prajapati et al. [11], the focus and novelty of this work is on coupling the anisotropic brittle crack model with the ductile crack model of Ambati et al. [16]. As modifications of Prajapati et al. [11], plastic deformations are additionally considered and the degradation function of Ambati et al. [16] is introduced to map ductile fracture characteristics. The calculation of the elastic and plastic fields is based on Herrmann et al. [22], and thus the mechanical jump conditions are applied to obtain locally homogenised stresses and plastic strains.

In Section 2, the model extension of the work presented by Prajapati et al. [11] is introduced in order to allow the propagation of cracks in anisotropic brittle and ductile materials. Section 3 discusses all relevant numerical aspects, while Section 4 includes numerical studies and applications. In Section 4.1, a detailed investigation of a crack is given, which passes a planar diffuse interface between an anisotropic brittle and a ductile phase. In Section 4.2, crack nucleation and propagation in a domain with a single anisotropic ellipsoid, embedded in a ductile matrix, is analysed. To show that important properties concerning the mechanical behaviour of grey cast iron can be predicted by the model, a simulation area, consisting of three elliptical, anisotropic inclusions, which are oriented differently in a ductile matrix, is subjected to tensile and compressive load in Section 4.3. Finally, Section 5 summarises the presented results and gives an outlook on upcoming applications.

## 2. Model Formulation

The model presented in Prajapati et al. [11] provides the basis for the present work. In order to allow simulations of crack nucleation and propagation in materials with anisotropic brittle and ductile constituents, the model is extended by an additional degradation function, which is sensitive to the accumulated plastic strain, as proposed by Ambati et al. [16]. The reasons for choosing this model from the numerous published ductile crack models are the following: In Ambati et al. [16], it is shown that important characteristics concerning ductile crack nucleation and propagation can be mapped by the model. This is made possible by a modified degradation function, which includes the accumulated plastic strain. The modification leads to crack initiation processes, which are consistent with experimental observations, despite the assumption of small deformations. Most of the features under consideration, e.g., the strain localisation in the middle of an I-shaped specimen, under tensile load, cannot be mapped by the majority of the other published ductile phase-field crack models. Compared to brittle phase-field crack models, the main difference in most other models that account for plastic deformations is that a plastic energy contribution, due to hardening, is introduced in the free energy functional, and that the stress calculation takes the plastic strains into account by splitting the total strain into an elastic and a plastic part [6]. These changes lead to stress–strain curves corresponding to a ductile material, but the plastic contribution in the energy functional, especially in the case of small deformations, is substantially smaller than the elastic strain energy and thus has only little influence on the crack nucleation and propagation. In Ambati et al. [16], the formulation of the ductile degradation function furthermore ensures that the experimentally determined yield points are represented accurately. If interested in alternative approaches of modelling ductile crack propagation using the phase-field method, the reader is referred to Alessi et al. [6]. In this work, popular phase-field models of fracture coupled with plasticity are compared by means of the resulting predictive capabilities for several well-defined problems.

In the classical understanding, phase-field models in which more than two phase fields can coexist are called multiphase-field models, which usually implies that each phase field possesses its own evolution equation. The existence of several evolution equations, however, only applies to the present model to a limited extent, since no solid-solid phase transformation is integrated. For this reason, the present model is not a full “multiphase model” but nevertheless builds on a multiphase-field representation of physically distinguishable regions and their interfaces. However, since a solid-solid phase evolution is to be integrated in further work and can be implemented straightforwardly, starting from the present model formulation, by applying a staggered scheme for the crack phase field and the solid-solid evolution, the solid-solid phase transitions were nevertheless discretised diffusely.

Within phase-field models, the transition between physically distinguishable solid phases is regularised, in order to allow an easy tracking of grain boundaries and to enable phase transformation processes. For this purpose, solid phase fields, $\phi^{\alpha }\left(x,t\right),\;\alpha =1\ldots N$, are introduced, with *N* as the number of solid phases. Within phase transition regions, the phase fields change continuously in the range of $0<\phi^{\alpha }<1$, while in material points, where only one phase α exists, $\phi^{\alpha }=1$ applies. To simplify the mathematical representation in the following, all solid phase fields are collected in the *N*-tuple $\hat{\phi }\left(x,t\right)$, and their gradients are joined in $\nabla \hat{\phi }\left(x,t\right)$.

Within phase-field crack models in a domain $\Omega \subset R^{d},d\in \left\{1,2,3\right\}$, the critical energy release rate is modelled in a regularised manner by a diffuse transition between solid phases and cracks, where by on top of the phase fields, representing the solid phases, an additional crack phase field, $\phi_{c}\left(x,t\right):\Omega \times R_{\geq 0}\to \left[0,1\right]$, is introduced in the spatial point x, at the time *t*. The crack phase field possesses a continuous transition between intact, $\phi_{c}=0$, and fractured, $\phi_{c}=1$, material points.

Each phase field represents the corresponding volume fraction of the phase, so that the constraint

(1) $$\begin{array}{c} \phi_{c}+\sum_{\alpha =1}^{N}\phi^{\alpha }=1 \end{array}$$

has to be fulfilled, which applies to the solid phase fields as well as the crack phase field. The understanding that each phase field reflects the volume fraction of a particular phase in a material point leads to the fact that during the growth of the crack phase field, i.e., the increase of the volume fraction of the crack phase, the corresponding volume fraction of the solid phases dissipates, which is comparable to continuum damage models. In terms of mass conservation, this is only acceptable as long as a negligible amount of the total mass of a system dissipates. If solid phases are converted into the crack phase, the relative composition of the solid phases is maintained with respect to the absolute volume fraction of the solid phases. Therefore, the transformation of the solid phase fields is given by

(2) $$\begin{array}{c} \dot{\phi^{\alpha }}\left(x,t\right)=-h_{s}^{\alpha }\dot{\phi_{c}}. \end{array}$$

Thus, it is sufficient to only solve the phase-field equation of the crack phase explicitly, into which the mechanical forces of a multiphase field problem are incorporated. To identify the relative volume fraction of a solid phase in relation to the absolute fraction of solid phases, it is required to formulate an interpolation function for solid phases:

(3) $$\begin{array}{c} h_{s}^{\alpha }\left(\hat{\phi }\right)=\frac{\phi^{\alpha }}{\sum_{\beta =1}^{N}\phi^{\beta }}, \end{array}$$

which satisfies the condition $\sum_{\alpha =1}^{N}h_{s}^{\alpha }=1$.

According to Griffith [7], in the case of linear fracture mechanics, crack growth occurs when the energy release rate of a material point, which corresponds to the elastic strain energy, exceeds the necessary free energy of an emerging free surface, given by a critical energy release rate $G_{c}\left(x,t\right)$. For the body Ω, this energetic view is captured in the phase-field context in a regularised way by using a free energy functional of the form

(4) $$\begin{array}{cc} F(\hat{\phi },\phi_{c},\nabla \phi_{c},\hat{\varepsilon_{e}},\varepsilon_{\mathrm{acc}}) & =\int_{\Omega }\underset{=f(\hat{\phi },\phi_{c},\nabla \phi_{c},\hat{\varepsilon_{e}},\varepsilon_{\mathrm{acc}})}{\underset{︸}{\frac{3}{8}\bar{G_{c}}(\frac{\phi_{c}}{\bar{l}}+\bar{l}{\left|\nabla \phi_{c}\right|}^{2})+f_{e}+f_{p}}}dV, \end{array}$$

which is comparable with [6,23], for example. The free energy functional includes the interpolated critical energy release rate, $\bar{G_{c}}$, the effective elastic strain energy,

(5) $$\begin{array}{c} f_{e}\left(\hat{\varepsilon_{e}},\varepsilon_{\mathrm{acc}},\hat{\phi }\right)=\sum_{\alpha =1}^{N}h_{s}^{\alpha }f_{e}^{\alpha }\left(\varepsilon_{e}^{\alpha },\varepsilon_{\mathrm{acc}}^{\alpha },\phi_{c}\right), \end{array}$$

and the effective plastic energy contribution, due to hardening,

(6) $$\begin{array}{c} f_{p}\left(\varepsilon_{\mathrm{acc}},\hat{\phi }\right)=\sum_{\alpha =1}^{N}h_{s}^{\alpha }f_{p}^{\alpha }\left(\varepsilon_{\mathrm{acc}}^{\alpha }\right), \end{array}$$

by applying the so-called AT-1 representation, according to [24], with regard to the geometrical shape of the diffuse transition between the solid phases and the crack. As a typing aid, all phase-inherent elastic tensors, $\varepsilon_{e}^{\alpha }$, are collected in $\hat{\varepsilon_{e}}$, and the phase-inherent accumulated plastic strains, $\varepsilon_{\mathrm{acc}}^{\alpha }$, are contained in $\varepsilon_{\mathrm{acc}}$. Phase-inherent quantities are quantities associated with each solid phase, i.e., each solid phase has its own mechanical fields. The width of the regularised transition between damaged and undamaged material points is determined by the interpolated regularisation parameter:

(7) $$\begin{array}{c} \bar{l}\left(\hat{l},\hat{\phi }\right)=\sum_{\alpha =1}^{N}h_{s}^{\alpha }l^{\alpha }. \end{array}$$

The regularisation parameter is considered as a material property and is chosen as proposed by Tanné et al. [25]. According to Tanné et al., the choice of the regularisation parameter guarantees that crack nucleation only takes place when the stresses in an isotropic brittle material reach the tensile strength. The calculation procedure for the determination of the regularisation parameter, shown in Tanné et al., is not readily applicable to the model used in this paper, which has an anisotropic and ductile material behaviour. Nevertheless, the correlations from Tanné et al. are used to calculate the regularisation parameters of each phase, $l^{\alpha }$, which are collected in $\hat{l}$, in order to use the best possible trade-off available. It is particularly important to mention that the regularisation parameters of transversely isotropic phases are related to the critical energy release rate and Young’s modulus of the weakest mechanical material direction.

It is known that the tension–compression asymmetry [26] of cast iron with lamellar graphite is controlled by the graphite lamellae [9,27]. While under tensile load, the graphite lamellae have a negligible loading capacity and act as microcracks. They tend to close under compressive forces and transmit the applied load [9,28]. To map this property, an unphysical crack propagation under compressive load must be avoided. Therefore, only the positive part of the phase-inherent elastic strain energy should lead to nucleation and growth of the crack phase. The elastic strain energy is decomposed into its positive and negative energy part:

(8) $$\begin{array}{c} f_{e}^{\alpha }\left(\varepsilon_{e}^{\alpha },\varepsilon_{\mathrm{acc}}^{\alpha },\phi_{c}\right)=h_{c}^{\alpha }{\left[f_{e}^{\alpha }\right]}^{+}+{\left[f_{e}^{\alpha }\right]}^{-}, \end{array}$$

using the spectral decomposition for isotropic phases, as proposed by Miehe et al. [4], and following Teichtmeister et al. [12], so as to allow a decomposition of transversely isotropic materials. Here, the crack degradation function $h_{c}^{\alpha }$ reflects the dissipation of the solid phases on the energetic level, caused by crack growth, and is applied exclusively to the positive energy contribution. To allow the transition of cracks between brittle and ductile phases, two different crack degradation functions are used:

(9) $$\begin{array}{c} h_{c}^{\alpha }\left(\varepsilon_{\mathrm{acc}}^{\alpha },\phi_{c}\right)=\{\begin{array}{cc} {\left(1-\phi_{c}\right)}^{2} & ,\;\mathrm{brittle}\\ {\left(1-\phi_{c}\right)}^{2\frac{\varepsilon_{\mathrm{acc}}^{\alpha }}{\varepsilon_{\mathrm{acc},\mathrm{crit}}^{\alpha }}} & ,\;\mathrm{ductile}. \end{array} \end{array}$$

The brittle degradation function is very common for brittle phase-field crack models. The ductile degradation function was introduced by Ambati et al. [16]. By incorporating the accumulated plastic strain into the ductile crack degradation function, the prediction of crack nucleation is possible, despite the small-strain formulation, which is in line with experimental findings [16]. Furthermore, it guarantees that the phase-inherent stresses of a ductile phase are not degraded, as long as no plastic deformation has occurred in a material point, even though the crack phase does exist. This ensures that plastic deformations only arise when the phase-inherent stress fulfils the yield condition. To control the ductile crack propagation, the threshold value $\varepsilon_{\mathrm{acc},\mathrm{crit}}\in R_{\geq 0}$ [16] is applied.

The local homogenisation approach of Herrmann et al. [22] is used as an elastoplastic multiphase-field model. Thus, the mechanical jump conditions of solid-solid phase transition regions are applied, which makes an interpolation of mechanical material parameters obsolete, apart from the critical energy release rate. The clean separation between elastoplastic and purely elastic phases is only possible without any difficulties, when this homogenisation approach is used. The homogenisation approach enables the calculation of phase-inherent stresses, $\sigma^{\alpha }$, and plastic strains, $\varepsilon_{p}^{\alpha }$, and therefore avoids the occurrence of plastic strains within brittle phases. Another advantage of the elastoplastic homogenisation approach is that an anisotropic elastic behaviour can be realised in one phase, even though an isotropic nonlinear constitutive model is applied in another phase. This holds especially in multiphase regions, which, in contrast, is not possible with models using interpolated material parameters. In multiphase regions, this is particularly required for the considered composite material, as the volumetrically interpolated stiffnesses of the isotropic pearlite and the transversely isotropic ellipsoids would not allow the $J_{2}$-plasticity model to be used in combination with a return mapping algorithm.

The following considerations are limited to small deformations; the total strain is expressed as $\varepsilon \left(H\right)=\frac{1}{2}\left(H+H^{\;T}\right)$, using the displacement gradient H=∇u(x,t); and an additive decomposition of the strains $\varepsilon =\varepsilon_{e}+\varepsilon_{p}$ is valid. The total, elastic, and plastic strains are identified as ε(x,t), $\varepsilon_{e}\left(x,t\right)$, and $\varepsilon_{p}\left(x,t\right)$. Within the simulations, sharp crack tips, caused by the brittle ellipsoids, act as stress concentrators. Thus, the local strains of >2% even occur when the macroscopic strain is small. Under compressive load, grey cast iron specimens fail at a macroscopic strain of >8%. Under the assumption of small deformations, the geometrical nonlinearities, which actually occur under the described states, are neglected for the sake of simplicity.

In material points with multiple solid phases, the mechanical jump conditions, given by the sharp interface theory of bounded solid-solid transitions [29], are used. On the one hand, this is the static balance of the linear momentum on a singular surface:

(10) $$\begin{array}{c} \left(\sigma^{\alpha }-\sigma^{\beta }\right)\;\cdot \;n^{\alpha \beta }=0, \end{array}$$

stating that the jump of the stresses of two phases α and β vanishes in the normal direction of a singular surface between the solid phases, $n^{\alpha \beta }\left(\nabla \hat{\phi }\right)$. On the other hand, the Hadamard jump condition

(11) $$\begin{array}{c} \left(H^{\alpha }-H^{\beta }\right)=a^{\alpha \beta }⊗n^{\alpha \beta } \end{array}$$

represents a no-slip condition in the tangential directions of singular surfaces. The rank-1 tensor $a^{\alpha \beta }$ defines the amplitude of the jump of the displacement gradient in the direction of the surface normal vector, as prescribed by the Hadamard lemma. In regions where only two phases coexist, the unit normal vectors between the two phases can be written as $n^{\alpha \beta }=\nabla \phi^{\alpha }/|\nabla \phi^{\alpha }|=-\nabla \phi^{\beta }/\left|\nabla \phi^{\beta }\right|$, according to [30]. In multiphase regions, the normal vector between two solid phases reads as $n^{\alpha \beta }=\left(\nabla \phi^{\alpha }-\nabla \phi^{\beta }\right)/\left|\nabla \phi^{\alpha }-\nabla \phi^{\beta }\right|$, as described in [31]. Applying the jump conditions with respect to the phase with the largest volume fraction, e.g., phase 1, in regions with *N* coexisting solid phases, N−1 unknown rank-1 tensors, $a^{12},a^{13},\ldots ,a^{1N}$, have to be determined to describe the jumps of the phase-inherent displacement gradients at the α–β transition in the $n^{\alpha \beta }$-direction. For the sake of simplicity, all rank-1 tensors are collected in the tuple

(12) $$\begin{array}{c} \hat{a}={\left(a^{12},a^{13},\ldots ,a^{1N}\right)}^{\;T}. \end{array}$$

The effective displacement gradient of multiphase material points, $H^{\mathrm{eff}}$, is defined by all phase-inherent displacement gradients, collected in $\hat{H}$, by means of a volumetric decomposition:

(13) $$\begin{array}{c} H^{\mathrm{eff}}\left(\hat{H},\hat{\phi }\right)=\sum_{\alpha =1}^{N}h_{s}^{\alpha }H^{\alpha }, \end{array}$$

where $H^{\alpha }$ are the phase-dependent displacement gradients. By rearranging the effective displacement gradient in combination with Equation (11), the displacement gradient of phase 1 can be written as

(14) $$\begin{array}{c} H^{1}=H^{\mathrm{eff}}+\sum_{\alpha =2}^{N}h_{s}^{\alpha }a^{1\alpha }⊗n^{1\alpha }. \end{array}$$

For the remaining deformation gradients, this results in

(15) $$\begin{array}{c} H^{\alpha }=H^{1}-a^{1\alpha }⊗n^{1\alpha }. \end{array}$$

All unknown rank-1 tensors, $a^{1\alpha }$, are calculated by solving the equation system

(16) $$\begin{array}{c} \hat{g}\left(\hat{a}\right)=(\begin{array}{c} (\sigma^{1}-\sigma^{2})\;\cdot \;n^{12}\\ (\sigma^{1}-\sigma^{3})\;\cdot \;n^{13}\\ \vdots \\ (\sigma^{1}-\sigma^{\tilde{N}})\;\cdot \;n^{1\tilde{N}} \end{array})=(\begin{array}{c} (h_{c}^{1}C^{1}[\varepsilon_{e}^{1}\left(\hat{a}\right)]-h_{c}^{2}C^{2}[\varepsilon_{e}^{2}\left(\hat{a}\right)])\;\cdot \;n^{12}\\ (h_{c}^{1}C^{1}[\varepsilon_{e}^{1}\left(\hat{a}\right)]-h_{c}^{3}C^{3}[\varepsilon_{e}^{3}\left(\hat{a}\right)])\;\cdot \;n^{13}\\ \vdots \\ (h_{c}^{1}C^{1}[\varepsilon_{e}^{1}\left(\hat{a}\right)]-h_{c}^{\tilde{N}}C^{\tilde{N}}[\varepsilon_{e}^{\tilde{N}}\left(\hat{a}\right)])\;\cdot \;n^{1\tilde{N}} \end{array})=0, \end{array}$$

composed of the pairwise static momentum balances on singular interfaces, Equation (10). The phase-inherent elastic strains are given by $\varepsilon_{e}^{\alpha }=\varepsilon^{\alpha }-\varepsilon_{p}^{\alpha }$, with $\varepsilon^{\alpha }=\frac{1}{2}(H^{\alpha }+{\left(H^{\alpha }\right)}^{\;T})$ taking Equations (14) and (15) into account. For the phase-inherent stresses, this results in

(17) $$\begin{array}{c} \sigma^{\alpha }=h_{c}^{\alpha }C^{\alpha }[\varepsilon_{e}^{\alpha }], \end{array}$$

where in the elastic stiffness tensor of phase α is denoted by $C^{\alpha }$. It can be observed that these stresses cannot be derived from the elastic strain energy, Equation (8), in the sense of a hyperelastic material model, since, unlike the elastic strain energy, the entire stress tensor is degraded, and not only its positive part. A similar approach to formulate a phase-field crack model was introduced in Ambati et al. [5], where this is referred to as a hybrid model.

A $J_{2}$-plasticity model [32] with linear isotropic hardening, resulting in the yield function

(18) $$\begin{array}{c} f_{y}^{\alpha }\left(s^{\alpha },\varepsilon_{\mathrm{acc}}^{\alpha }\right)=\sqrt{\frac{3}{2}}\left|s^{\alpha }\right|-\left(\sigma_{y,0}^{\alpha }+H^{\alpha }\varepsilon_{\mathrm{acc}}^{\alpha }\right)\leq 0, \end{array}$$

is applied to compute the phase-inherent plastic strains $\varepsilon_{p}^{\alpha }$. Here, $s^{\alpha }=\sigma^{\alpha }-1/3\mathrm{tr}\left(\sigma^{\alpha }\right)I$ represents the deviatoric stress tensor, and $|s^{\alpha }|=\sqrt{s_{ij}^{\alpha }s_{ij}^{\alpha }}$ its Frobenius norm, where the Einstein summation convention is valid. The first term of the yield function is the equivalent von Mises stress, which is compared with the yield stress, expressed by the yield strength $\sigma_{y,0}^{\alpha }$ and the strain hardening $H^{\alpha }\varepsilon_{\mathrm{acc}}^{\alpha }$, which includes the phase-inherent linear hardening parameter $H^{\alpha }$. If the yield function is violated, plastic yielding occurs. Using the associative flow rule

(19) $$\begin{array}{c} {\dot{\varepsilon }}_{p}^{\alpha }={\dot{\gamma }}^{\alpha }\frac{\partial f_{y}^{\alpha }\left(s^{\alpha },\varepsilon_{\mathrm{acc}}^{\alpha }\right)}{\partial s^{\alpha }}, \end{array}$$

the evolution of the plastic strain is described. The phase-inherent accumulated plastic strain $\varepsilon_{\mathrm{acc}}^{\alpha }=\int_{t}\sqrt{2/3}\left|{\dot{\varepsilon }}_{p}^{\alpha }\right|dt$ serves as an internal variable. As a result, the internal plastic energy contribution of phase α [32] reads as

(20) $$\begin{array}{c} f_{p}^{\alpha }\left(\varepsilon_{\mathrm{acc}}^{\alpha }\right)=\frac{1}{2}H^{\alpha }{\varepsilon_{\mathrm{acc}}^{\alpha }}^{2}. \end{array}$$

To establish the consistency condition $f_{y}^{\alpha }\overset{!}{=}0$ in the case of plastic yielding, a two-step return mapping algorithm is employed. Applying a volumetric interpolation, the effective accumulated plastic strain

(21) $$\begin{array}{c} \varepsilon_{\mathrm{acc}}^{\mathrm{eff}}\left(\varepsilon_{\mathrm{acc}},\hat{\phi }\right)=\sum_{\alpha =1}^{N}h_{s}^{\alpha }\varepsilon_{\mathrm{acc}}^{\alpha } \end{array}$$

is introduced.

To solve the static balance of linear momentum,

(22) $$\begin{array}{c} \mathrm{div}\sigma^{\mathrm{eff}}=0, \end{array}$$

for the displacement field u(x,t), the degraded volume-averaged effective stresses

(23) $$\begin{array}{c} \sigma^{\mathrm{eff}}\left(\hat{\sigma },\hat{\phi }\right)=\sum_{\alpha =1}^{N}h_{s}^{\alpha }\sigma^{\alpha } \end{array}$$

are used, which result from all phase-inherent stresses, collected in $\hat{\sigma }$.

An important part of a phase-field crack model with diffusely overlapping transition regions of solid phases is the volumetrically interpolated critical energy release rate

(24) $$\begin{array}{c} \bar{G_{c}}\left(\hat{\phi },\nabla \phi_{c}\right)=\sum_{\alpha =1}^{N}h_{s}^{\alpha }G_{c}^{\alpha }, \end{array}$$

allowing the transition between phases with different crack characteristics and anisotropy orientations. A transverse anisotropy resulting for, e.g., lamellar graphite from its atomistic structure, as indicated in the introduction, can be modelled for phase α, with an anisotropic critical energy release rate, such as

(25) $$\begin{array}{c} G_{c}^{\alpha }\left(\nabla \phi_{c}\right)=G_{c,0}^{\alpha }({\left({\tilde{n}}_{x}^{\alpha }\right)}^{2}+F_{\mathrm{aniso}}^{\alpha }({\left({\tilde{n}}_{y}^{\alpha }\right)}^{2}+{\left({\tilde{n}}_{z}^{\alpha }\right)}^{2})). \end{array}$$

Through the phase-inherent anisotropy factor $F_{\mathrm{aniso}}^{\alpha }\in R_{>=1}$, the referential critical energy release rate $G_{c,0}^{\alpha }$ is deformed into an ellipsoid. If $F_{\mathrm{aniso}}^{\alpha }=1$ is chosen, the critical energy release rate is isotropic, which is the case for ductile phases. The orientations of the phase-dependent anisotropies are described by the phase-inherent Euler angles and are taken into account by the transformation matrices $Q_{\mathrm{aniso}}^{\alpha }\left(\varphi_{\mathrm{aniso}}^{\alpha },\theta_{\mathrm{aniso}}^{\alpha },\psi_{\mathrm{aniso}}^{\alpha }\right)$, which are applied to transform the outward-pointing normal vector of the crack phase field from the reference x,y,z-coordinate system into the hexagonal lattice $\tilde{x},\tilde{y},\tilde{z}$-coordinate systems of graphite lamellae:

(26) $$\begin{array}{c} {\tilde{n}}^{\alpha }\left(\nabla \phi_{c}\right)=(\begin{array}{c} {\tilde{n}}_{x}^{\alpha }\left(\nabla \phi_{c}\right)\\ {\tilde{n}}_{y}^{\alpha }\left(\nabla \phi_{c}\right)\\ {\tilde{n}}_{z}^{\alpha }\left(\nabla \phi_{c}\right) \end{array})=-Q_{\mathrm{aniso}}^{\alpha }n_{c}=-Q_{\mathrm{aniso}}^{\alpha }\frac{\nabla \phi_{c}}{|\nabla \phi_{c}|}. \end{array}$$

Graphite lamellae show a preferred growth direction along their graphene planes (a-direction of their hexagonal crystal system) [33]. Therefore, their spatial expansion within the basal planes is much larger than orthogonal to it [34]. Based on this fact, the orientation of the longitudinal axis of a graphite lamella can be used to define its transformation matrices $Q_{\mathrm{aniso}}^{\alpha }$. From the formulation of Equation (25), it can be concluded that the crystalline a-direction of the hexagonal crystal system of a graphite lamella points in the same direction as the $\tilde{x}$-axis.

Following Allen-Cahn [35], the temporal and spatial evolution of the crack phase field is given as

(27) $$\begin{array}{c} \dot{\phi_{c}}\left(x,t\right)=-M\frac{\delta F(\hat{\phi },\phi_{c},\nabla \phi_{c},\hat{\varepsilon_{e}},\varepsilon_{\mathrm{acc}})}{\delta \phi_{c}}, \end{array}$$

including the variational derivative of the total free energy of the system, with respect to the crack phase field and the kinetic coefficient *M*. To correctly predict the kinetics of crack propagation, the kinetic coefficient has to be chosen according to experimental findings. In this work, the explicit Euler scheme is applied to calculate the time derivatives in order to determine an equilibrium state of the crack phase field for each mechanical load increment, $\dot{\phi_{c}}\approx 0$. Thus, there is only a fictive time dependence, and the kinetic coefficient can be chosen in such a way that the simulations are numerically stable. The performed variational derivation of Equation (4) results in

(28) $$\begin{array}{cc} \dot{\phi_{c}}= & -M(\frac{\partial f}{\partial \phi_{c}}-\nabla \;\cdot \;\frac{\partial f}{\partial \nabla \phi_{c}})\\ = & -M[\underset{g_{c}\left(\hat{\phi },\phi_{c},\nabla \phi_{c},\hat{\varepsilon_{e}},\varepsilon_{\mathrm{acc}}\right)\leq 0}{\underset{︸}{\frac{3}{8}(\bar{G_{c}}(\frac{1}{\bar{l}}-2\bar{l}\nabla \;\cdot \;\nabla \phi_{c})-\nabla \;\cdot \;\frac{\partial \bar{G_{c}}}{\partial \nabla \phi_{c}}(\frac{\phi_{c}}{\bar{l}}+\bar{l}{\left|\nabla \phi_{c}\right|}^{2}))+\frac{\partial f_{e}}{\partial \phi_{c}}}}], \end{array}$$

where by

(29) $$\begin{array}{c} \frac{\partial n_{c}\left(\nabla \phi_{c}\right)}{\partial \nabla \phi_{c}}=\frac{I-n_{c}⊗n_{c}}{|\nabla \phi_{c}|} \end{array}$$

applies for the derivation of the normal vector of the crack phase field [36], which is necessary for the calculation of $\partial \bar{G_{c}}/\partial \nabla \phi_{c}$. It should be considered that all vanishing terms of the variational derivative are already excluded in the equation above. To prevent crack healing, $\dot{\phi_{c}}\geq 0$ is enforced. Therefore, the presented model is classified as a damage model, as the evolution of the crack phase field is irreversible.

All governing equations of the crack model are summarised in Table 1. The meaning of the symbols is summarized in Table 2.

### Model Restrictions

The starting point of developing this model was to study the evolution of cracks in cast iron with lamellar graphite. However, this is only possible to a limited extent, as the new model cannot be applied to the actual length scale, the micrometer range, necessary to resolve the microstructure of cast iron with lamellar graphite. However, with the presented model, the step towards a model that is applicable on the microscopic length scale is no longer far off. As an adjustment, only one extension is necessary, so that the model formulation is independent of the choice of the parameter to define the width of the diffuse transition between the solid phases and the cracks. This could be done in a similar way as the procedure published by Wu and Nguyen [37], but with the additional difficulty that instead of a brittle-isotropic model formulation, a brittle-anisotropic model formulation, combined with a ductile formulation, would have to be considered.

## 3. Numerical Aspects

In this section, a brief overview of the numerical treatment is provided, which is applied in this publication.

All simulations were executed with the in-house software package Pace3D [38] (Parallel Algorithms for Crystal Evolution in 3D). The equations are implemented for general 3D problems, although simulations in quasi-2D domains, with one cell in *z*-direction of space, were performed exclusively, due to computational efforts. An equidistant orthogonal grid is used, since curvatures can be well approximated, using a diffuse transition between different phases. For the mapping of curvatures, a more complex discretisation grid is not necessary. Initially, a diffuse interface is established between all occurring solid phases. The calculation of the phase fields and the mechanical fields is processed in a staggered manner, which means that no monolithic solution is used, where the fields serve as an input to each other and are interpolated into the required positions of the discrete cells.

The phase-field Equation (27) is solved at central positions of the numerical cells by applying the explicit Euler scheme for the time derivative and the finite-difference method (FDM), using second-order accurate central differences for the spacial derivatives. If, due to the explicit Euler scheme, values of the crack phase field occur that are greater than one, all solid phase fields are set to zero, and $\phi_{c}=1$ holds. In points where the gradient of the crack phase field is very smaller, isotropic material behaviour of the critical energy release rate, $F_{\mathrm{aniso}}^{}=1$, is assumed. This is done to avoid numerical inaccuracies in the calculation of normal vectors.

On the discretisation grid, the positions of the stresses and strains correspond to an FE mesh with linear elements and a full integration. Using a predictor-corrector two-step return mapping scheme, the displacement fields are calculated locally with respect to the mechanical jump conditions (see Section 2). The global adjustment of the displacement fields is done by a Newton–Raphson algorithm. First, initial predictor displacement fields are determined iteratively, fulfilling the mechanical equilibrium condition, given by Equation (22), including the plastic strains of the previous time step/initial values. After the calculation of the unknown plastic strains and the accumulated plastic strains, based on the elastic prediction, the mechanical equilibrium condition might be violated. From the violated mechanical equilibrium condition, a correction of the displacement fields is calculated and applied to the current displacement fields. This procedure describes one iteration step of the global Newton scheme and is repeated until the mechanical equilibrium condition holds for the new plastic strains.

For each load increment of the mechanical boundary conditions, the phase fields are brought into a steady state. Using fixed mechanical boundary conditions and constant mechanical fields, the phase-field evolution Equation (27) is solved explicitly for constant normal vectors of the crack phase field, until the criterion $\dot{\phi_{c}}\approx 0$ is fulfilled. After fulfilling the equilibrium condition, updated normal vectors of the crack phase field are calculated. As soon as the steady state of the phase fields holds for the updated normal vectors, the mechanical load is increased. Through the described procedure, the time increment Δt and the kinetic coefficient *M* of the phase-field evolution Equation (27) have only a negligible influence on the simulation results and are chosen in such a way that no numerical instabilities occur. Since the simulation results are time-independent, it is only possible to make conclusions about possible crack paths, but not about the kinetics of the crack growth.

## 4. Numerical Examples

In this section, the developed model is validated and verified. As the anisotropic brittle crack model has already been validated in our previous publication Prajapati et al. [11], and the ductile crack model for single solid phases in Ambati et al. [16], the focus of the following examinations is on the crack nucleation and propagation in domains with brittle, transversely isotropic and ductile solid phases. Section 4.1 discusses an initial crack, passing the planar diffuse interface of a brittle, transversely isotropic and ductile solid phase. In Section 4.2, the crack nucleation and propagation of a domain with a single brittle, transversely isotropic ellipsoid, which imitates a graphite lamella, embedded in a pearlitic matrix is analysed. Finally, crack nucleation and growth are simulated in a simulation area consisting of three differently oriented elliptical inclusions, embedded in a ductile matrix, which is subjected to tensile and compressive load. The simulation areas are assumed to have a physical size of 80 mm × 80 mm in order to ensure the correct use of the regularisation parameter between the solid phases and crack, where Δx=Δy=Δz=0.265 mm is chosen as grid spacing.

The model allows the simulation of combined anisotropic and ductile properties of a single phase, which, however, is not necessary for the fictive materials under consideration, since the imitated graphite lamellae are anisotropic and brittle, while the pearlitic matrix is isotropic and ductile. In the present study, a combination of anisotropic and ductile properties is not considered.

Table 3 summarises the applied material parameters for the performed simulations. Young’s modulus and the fracture toughness of the graphite lamellae are taken from Pickup et al. [39], while the Poisson’s ratio is given by Fishlock et al. [40]. The pearlitic material parameters are standard values for steel. Through the introduced material parameters, the critical energy release rate $G_{c}$ is given as $G_{c}=K_{\mathrm{Ic}}^{2}\left(1-\nu^{2}\right)/E$, according to a plane strain state. For the calculation of the phase-dependent regularisation parameters $l^{\alpha }$, according to Tanné et al. [25], tensile strengths from Zhang et al. [41] and Boyer [42] are used.

Transversely isotropic material stiffnesses are obtained with the help of Young’s modulus, Poisson’s ratio, and the anisotropy factor of Equation (25). Considering the atomic structure of graphite, made from many layered basal planes, a transversally isotropic stiffness tensor of the following form is obtained within the hexagonal lattice $\tilde{x},\tilde{y},\tilde{z}$-coordinate systems of graphite lamellae:

(30) $$\begin{array}{c} C_{\mathrm{tran}.}={\left(\begin{array}{cccccc} C_{22}\left(1+F_{\mathrm{aniso}}^{}\right) & \lambda & C_{12}\left(1+F_{\mathrm{aniso}}^{}\right) & 0 & 0 & 0\\ \; & \lambda +2\mu & C_{12} & 0 & 0 & 0\\ \; & \; & C_{22}\left(1+F_{\mathrm{aniso}}^{}\right) & 0 & 0 & 0\\ \; & \mathrm{sym}. & \; & \frac{C22-C12}{2} & 0 & 0\\ \; & \; & & \; & \frac{C11-C13}{2} & 0\\ \; & \; & & \; & & C_{44} \end{array}\right)}_{\tilde{x},\tilde{y},\tilde{z}}. \end{array}$$

Herein, the Lamé constants are given by

(31) $$\begin{array}{c} \lambda =\frac{\nu E}{\left(1-2\nu )(1+\nu \right)};\;\mu =\frac{E}{2(1+\nu )}. \end{array}$$

Again, it is assumed that the basal planes, pointing in the a-direction of the hexagonal crystal system, are parallel to the $\tilde{x},\tilde{z}$-plane, as in the case of the anisotropic critical energy release rate (25). The procedure used here achieves a correlation between the anisotropy strength of the critical energy release rate and the material stiffness.

Each simulation was carried out with the same mechanical boundary conditions, which are shown in the following Section 4.1. The boundaries in *x*-direction are defined as stress-free. On the lower and upper boundaries of the domain, an orthogonal displacement *u* is applied in an incremental manner. The orthogonal displacement boundary condition allows a free contraction in directions tangential to normal vectors of free surfaces. To create a condition of the quasi-2D domain corresponding to a plane strain state, the orthogonal displacement boundary condition is also applied in the *z*-direction, where the orthogonal displacements are equal to zero. After the condition $\dot{\phi_{c}}<10^{-4}$ holds for the updated normal vectors of the crack phase field, the mechanical load is increased.

The visualisation of the crack phase field $\phi_{c}$ is done by overlaying the solid phases and a continuous transition from opaque (black), $\phi_{c}=1$, to transparent, $\phi_{c}=0$.

### 4.1. Planar Interface between Brittle-Anisotropic and Ductile Solid Phase

**Figure 1:**
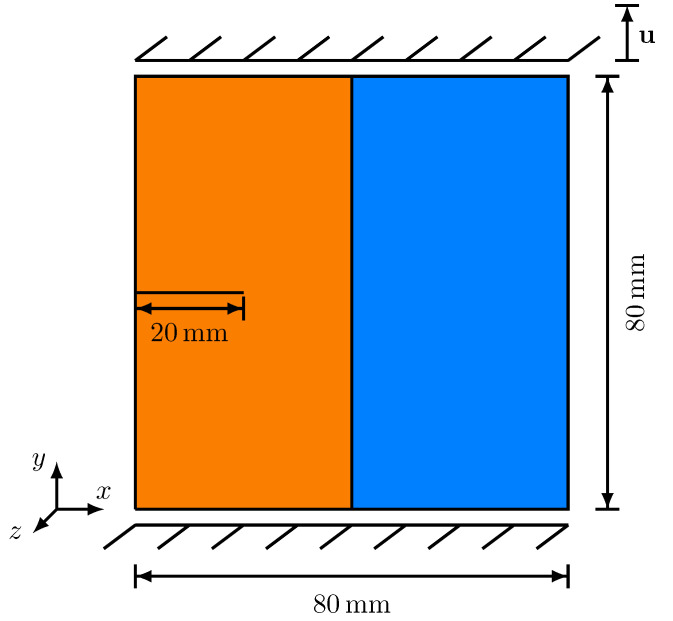
Simulation domain for a crack passing a vertical planar diffuse interface, accompanied by the mechanical boundary conditions. The dotted lines indicate the diffuse transition between the two solid phases.

The investigated problem consists of the transition of an anisotropic brittle phase into a ductile phase, by means of a planar interface, where by the left half of the domain corresponds to the anisotropic brittle phase and the right half to the ductile phase. On the left side of the simulation domain, a pre-existing 20 mm long initial crack is located. In Figure 1, the simulation setup and the mechanical boundary conditions are depicted. Only uniaxial tensile tests were carried out, whose mechanical boundary conditions are specified at the beginning of this Section 4.

In this example, both phases use the material properties of pearlite (Table 3) to ensure transcrystalline crack growth. However, the brittle phase still makes use of the transversely isotropic stiffness tensor (Equation (30)) with no plastic deformations occurring.

In the following studies, only the influence of a single parameter is examined at once. Therefore, the following set of standard model parameters is defined, which is kept constant, while one parameter is varied:

(32) $$\begin{array}{c} \epsilon =3\Delta x;\;\varepsilon_{\mathrm{acc},\mathrm{crit}}^{d}=0.08;\;F_{\mathrm{aniso}}^{b}=2;\;\psi_{\mathrm{aniso}}^{b}=30{}^{\circ}. \end{array}$$

The influences of the following parameters on the crack propagation and the homogenised macroscopic stress–strain response are investigated: the parameter ϵ, which defines the width of the diffuse transition between the solid phases; the rotation angle $\psi_{\mathrm{aniso}}^{b}$; and the anisotropy factor $F_{\mathrm{aniso}}^{b}$ of the transverse isotropy around the *z*-axis of the reference x,y,z-coordinate system, as well as the threshold value $\varepsilon_{\mathrm{acc},\mathrm{crit}}^{d}$ of the ductile phase.

**Figure 2:**
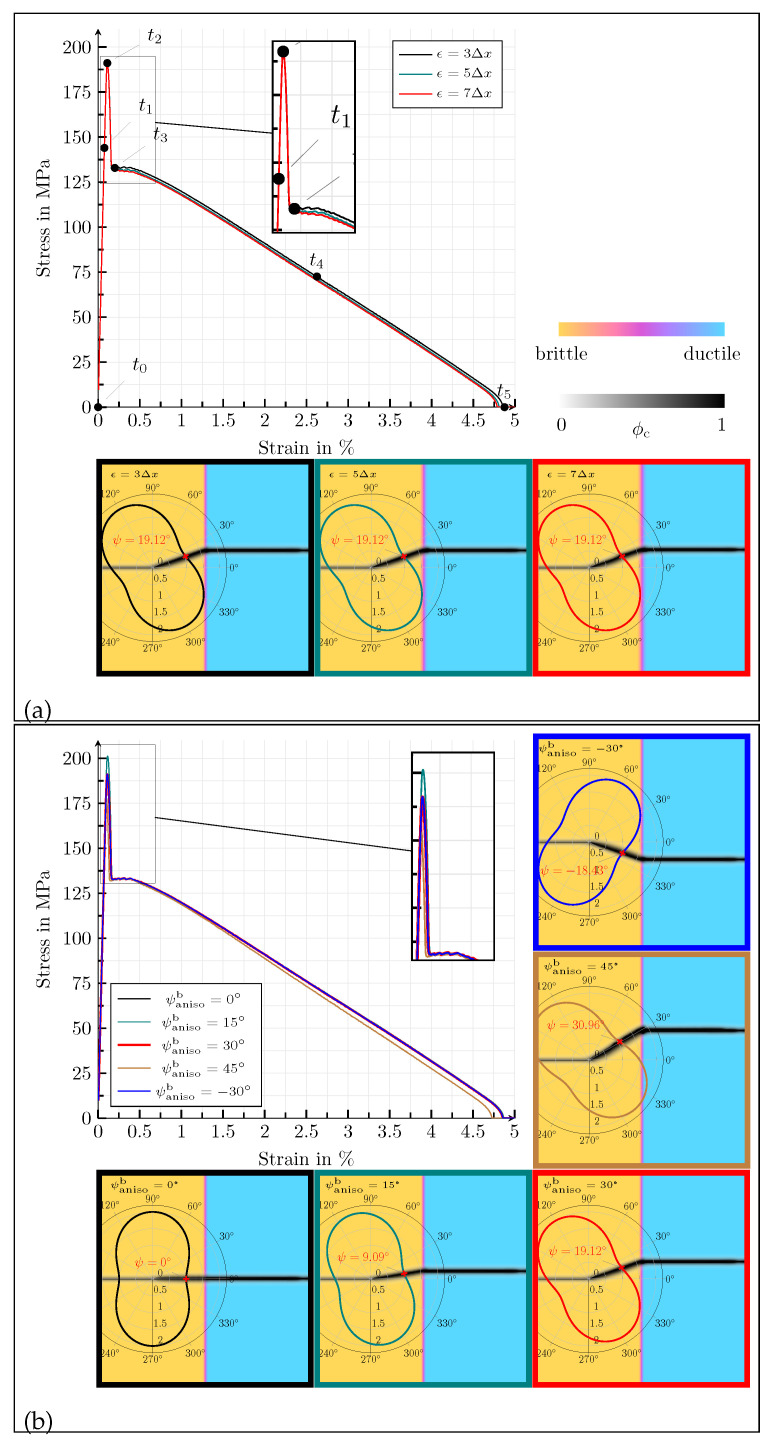
(**a**) Homogenised macroscopic stress–strain responses in the case of uniaxial tension for different values of *ϵ* = {3, 5, 7}Δ*x*, for the simulation setup with a planar diffuse interface between two solid phases. The dots in the stress–strain diagram correspond to the strain states of the contour plots of Figure 3. Below the diagram, contour plots of the crack phase $\phi_{c}$ at fracture are given, overlaying the solid phases for the various values of ϵ. (**b**) Homogenised macroscopic stress–strain responses in the case of uniaxial tension for different values of $\psi_{\mathrm{aniso}}^{b}=$ {0, 15, 30, 45, −30}°, for the simulation setup with a planar diffuse interface between two solid phases. The contour plots of the crack phase $\phi_{c}$ at fracture, overlaying the solid phases, for the different values of $\psi_{\mathrm{aniso}}^{b}$ are given below and beneath the stress–strain diagram.

**Figure 3:**
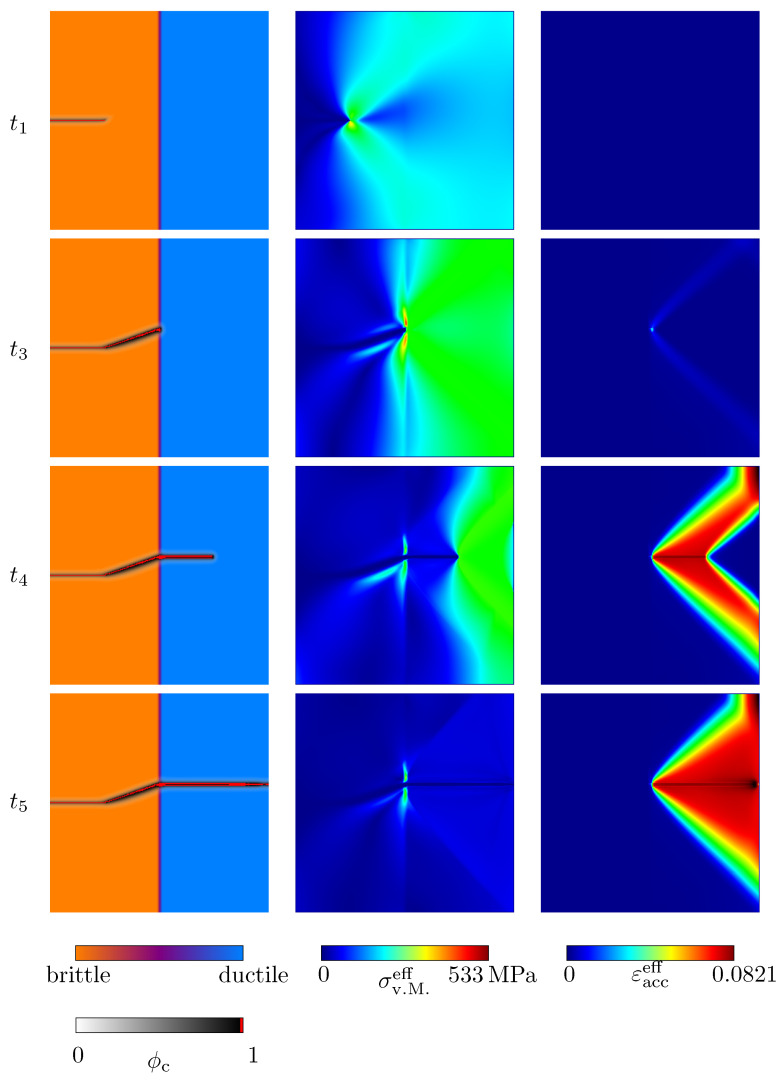
Contour plots of the evolution of the crack phase field $\phi_{c}$ (**first column**) and the resulting von Mises stresses $\sigma_{v.M.}^{\mathrm{eff}}$ (**second column**), together with the effective accumulated plastic strain $\varepsilon_{\mathrm{acc}}^{\mathrm{eff}}$ (**third column**) for the simulation setup with a planar diffuse interface between two solid phases for the set of standard parameters, Equation (32). The corresponding macroscopic uniaxial strain states are given in Figure 2a.

First of all, the influence of the length parameter for the definition of the width of the diffuse solid-solid phase transitions is investigated for the values *ϵ* = {3, 5, 7}Δ*x*, in terms of the homogenised macroscopic stress–strain response and the crack morphology at fracture (see Figure 2a). The corresponding contour plots of the equivalent von Mises stresses of Equation (23), $\sigma_{v.M.}^{\mathrm{eff}}$, and the effective accumulated plastic strain, $\varepsilon_{\mathrm{acc}}^{\mathrm{eff}}$, for the highlighted states in Figure 2a, as well as the set of standard parameters, Equation (32), are shown in Figure 3. It can be observed that a linear elastic range is present in the macroscopic stress–strain diagrams ($t_{0}$–$t_{2}$) before the stress value drops abruptly after its peak ($t_{2}$), in the same manner as brittle materials, without showing a macroscopic plastic deformation. The steep descent ($t_{2}$–$t_{3}$) is caused by an unstable crack growth within the brittle anisotropic phase. As can be seen in the polar plots, overlaying the final crack paths in Figure 2a, the angle between the crack in the anisotropic phase and the *x*-axis of the reference coordinate system is ψ=19.2°. The discrepancy between the inclination of the crack and the orientation of the anisotropy occurs because the elastic strain energy tends to target a perfectly horizontal crack (ψ=0°) when exposed to a load of Mode-I, whereas the formulation of the critical energy release rate forces a slope, corresponding to $\psi_{\mathrm{aniso}}^{b}$. These two counterparts cause an actual inclination of the crack between ψ=0° and $\psi_{\mathrm{aniso}}^{b}$ [11]. As soon as the crack reaches the diffuse interface between the solid phases, the characteristics of the macroscopic stress–strain plots and the crack morphology change. The further course of the macroscopic stress–strain response is strongly nonlinear ($t_{4}$) and is associated with plastic deformations. The crack path loses its inclination and spreads horizontally in a pure Mode-I manner. After the sample is broken ($t_{5}$), the macroscopic stress drops to zero, while on the level of the contour plots, residual stresses remain locally, caused by the plastic deformations. In Figure 2a, it can be seen that the width of the diffuse transition between the solid phases has almost no influence on the macroscopic stress–strain curve and the final crack morphology in the considered range for ϵ.

Next, the anisotropy orientation for the values $\psi_{\mathrm{aniso}}^{b}=$ {0, 15, 30, 45, −30}° is varied. The corresponding stress–strain curves and the final crack paths are depicted in Figure 2b. Due to the anisotropic stiffness tensor of the brittle phase, the peak stress value of the diagram increases with an increasing anisotropy angle $\psi_{\mathrm{aniso}}^{b}$. In analogy to graphite, only the van der Waals forces are stressed at a rotation angle of $\psi_{\mathrm{aniso}}^{b}=0{}^{\circ}$, whereas only the covalent bonds are stressed at $\psi_{\mathrm{aniso}}^{b}=90{}^{\circ}$, resulting in higher macroscopic stress values. As the rotation angle of the anisotropy increases, the deflection of the resulting crack also increases as expected, where by it should again be noted that the anisotropic formulation and the strain energy act as opponents. When comparing $\psi_{\mathrm{aniso}}^{b}=30{}^{\circ}$ and $\psi_{\mathrm{aniso}}^{b}=-30{}^{\circ}$ in Figure 2b, it can be observed that the resulting stress–strain diagrams for a change of sign of the rotation angle are almost equivalent. The small differences that occur are due to numerical inaccuracies that arise because the mechanical load is only applied at the upper edge of the simulation area. For varying orientations of anisotropy, the ductile part of the stress–strain curves does not differ significantly; all curves are nearly identical, except for $\psi_{\mathrm{aniso}}^{b}=45{}^{\circ}$. Due to geometric reasons, the cracks become wider with increasing deflection within the brittle phase, as they pass through the solid phase transition region. This phenomenon has an effect on the stress–strain curve of the simulation with an anisotropy orientation of $\psi_{\mathrm{aniso}}^{b}=45{}^{\circ}$.

**Figure 4:**
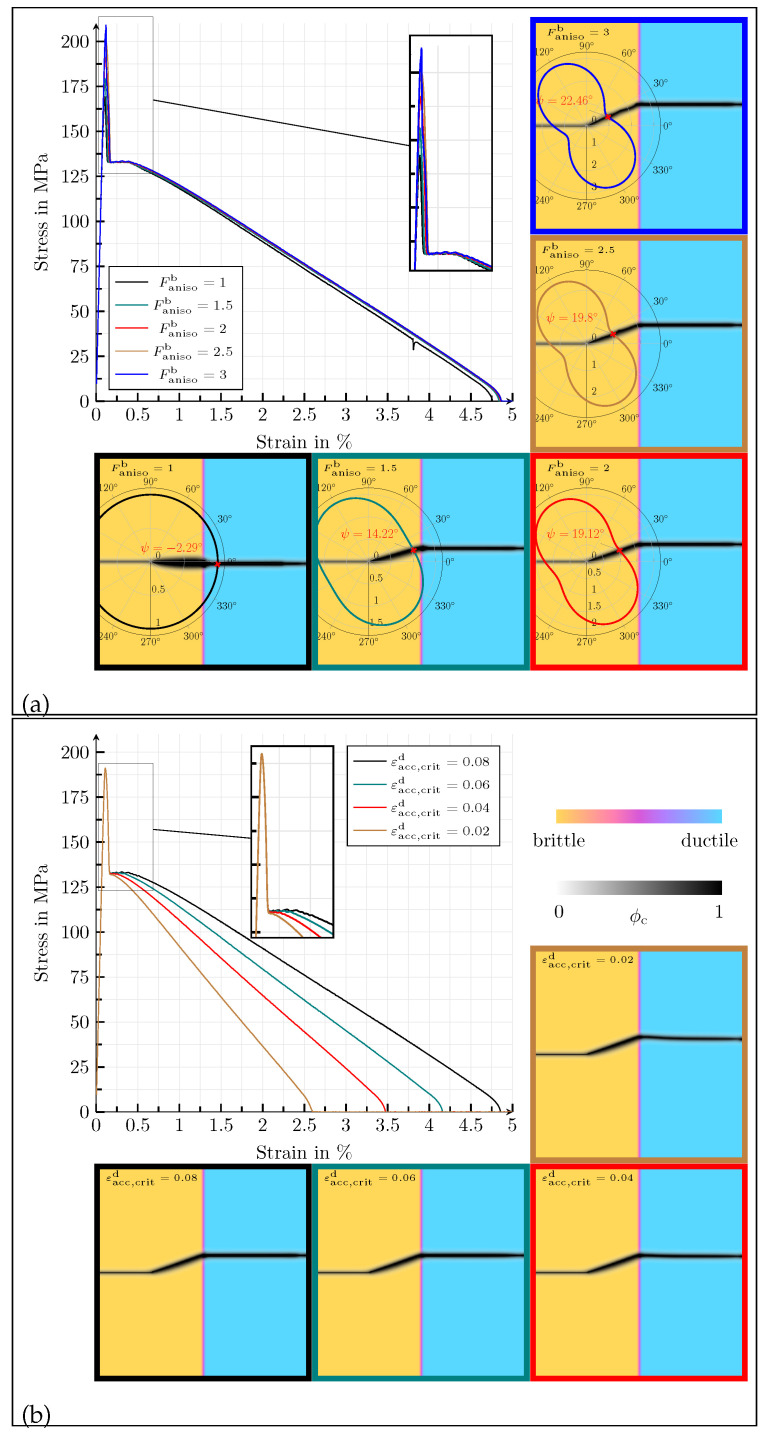
(**a**) Homogenised macroscopic stress–strain responses in the case of uniaxial tension for different values of $F_{\mathrm{aniso}}^{b}=$ {1, 1.5, 2, 2.5, 3}, for the simulation setup with a planar diffuse interface between two solid phases. The contour plots of the crack phase $\phi_{c}$ at fracture, overlaying the solid phases, for the different values of $F_{\mathrm{aniso}}^{b}$ are given below and beneath the stress–strain diagram. (**b**) Homogenised macroscopic stress–strain responses in the case of uniaxial tension for different values of $\varepsilon_{\mathrm{acc},\mathrm{crit}}^{d}=$ {0.08, 0.06, 0.04, 0.02}, for the simulation setup with a planar diffuse interface between two solid phases. The contour plots of the crack phase $\phi_{c}$ at fracture, overlaying the solid phases, for the different values of $\varepsilon_{\mathrm{acc},\mathrm{crit}}^{d}$ are given below and beneath the stress–strain diagram.

In Figure 4a, the influence of the anisotropy factor on the mechanical behaviour is discussed for the values $F_{\mathrm{aniso}}^{b}=$ {1,  1.5, 2, 2.5, 3}. The stress–strain curve shows higher stress peaks as the anisotropy factor increases and thus also the effective stiffness of the simulation domain. The reason for this is that the anisotropy factor increases the stiffness of the brittle phase. In analogy to lamellar graphite, the increase in the elastic modulus occurs within the basal planes along the a-direction of the hexagonal crystal system. If the anisotropy factor is chosen as $F_{\mathrm{aniso}}^{b}=1$, the brittle phase behaves isotropically, and the crack profile has no slope, ψ=0°. The deflection of the crack increases with increasing anisotropy factor, due to the anisotropic critical energy release rate.

Finally, the influence of the threshold value $\varepsilon_{\mathrm{acc},\mathrm{crit}}^{d}$ of the ductile phase is investigated for the values $\varepsilon_{\mathrm{acc},\mathrm{crit}}^{d}=$ {1,  0.6, 0.4, 0.2}. The corresponding results are given in Figure 4b. As long as the crack propagates within the brittle phase, a change of the threshold value has no influence on the material behaviour, neither with regard to the stress–strain behaviour nor with regard to the crack morphology. In the ductile phase, the crack morphology is also not significantly altered by the threshold value. However, as expected, there is a strong influence on the course of the stress–strain diagram. As the threshold increases, the elongation until complete failure rises, because the ductile degradation function depends on $\varepsilon_{\mathrm{acc},\mathrm{crit}}^{d}$, which means that higher plastic deformations are required to cause crack propagation in the ductile phase as $\varepsilon_{\mathrm{acc},\mathrm{crit}}^{d}$ increases.

### 4.2. Single Elliptic, Transversely Isotropic Brittle Inclusion in Ductile Matrix

**Figure 5:**
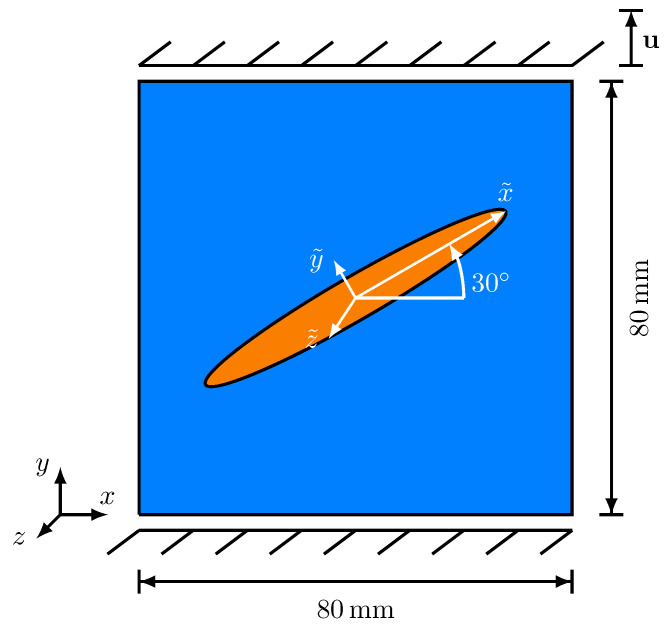
Setup of the crack development investigation in a domain consisting of an elliptic, transversely isotropic brittle inclusion, embedded in a ductile matrix, accompanied by the mechanical boundary conditions. The ductile matrix is represented in blue, while the brittle inclusion phase is represented in orange.

In this section, a single elliptic, transversely isotropic brittle inclusion, embedded in a ductile matrix, is used as a simulation setup. The longitudinal axis of the ellipsoid, which is equivalent to the $\tilde{x}$-direction of the anisotropic critical energy release rate, is rotated with respect to the *z*-axis of the reference coordinate system. Its length is 64 mm and its width 4 mm. In Figure 5, the simulation setup and the mechanical boundary conditions are depicted. For the uniaxial tensile tests, the mechanical boundary conditions, introduced at the beginning of this Section 4, are applied.

As material parameters for the anisotropic brittle phase, the values of graphite given in Table 3 are used and applied to Equation (30). The ductile matrix possesses the mechanical properties of pearlite as given in Table 3. In the following investigations, only the influence of one parameter is examined at a time, as was done previously. Therefore, the set of standard model parameters, Equation (32), is used, which is kept constant in case another parameter is varied.

**Figure 6:**
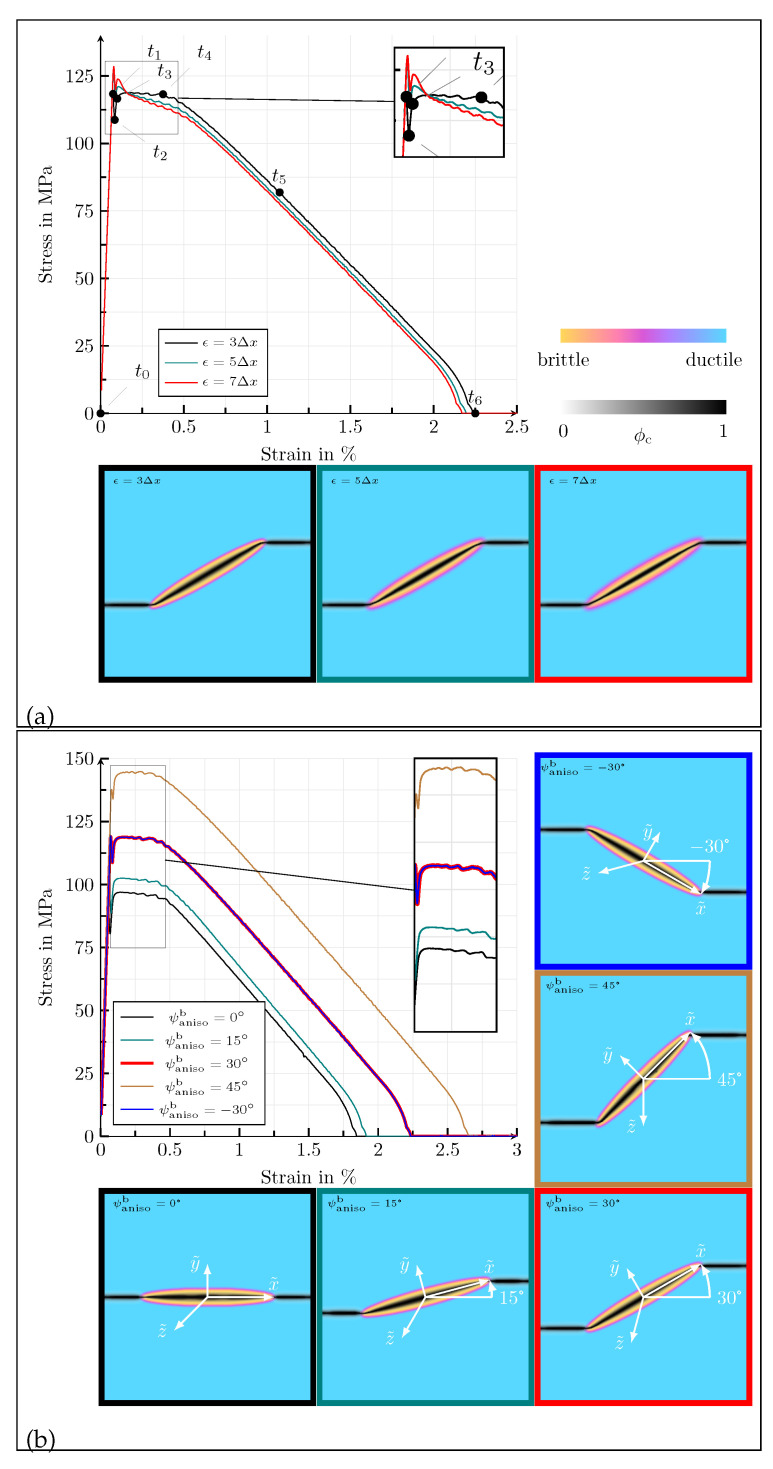
(**a**) Homogenised macroscopic stress–strain responses in the case of uniaxial tension for different values of *ϵ* = {3, 5, 7}Δ*x*, for the simulation setup with a single elliptic, transversely isotropic, brittle inclusion in a ductile matrix. The dots in the stress–strain diagram correspond to the strain states of the contour plots of Figure 7. Below the diagram, contour plots of the crack phase $\phi_{c}$ at fracture are given, overlaying the solid phases for the various values of ϵ. (**b**) Homogenised macroscopic stress–strain responses in the case of uniaxial tension for different values of $\psi_{\mathrm{aniso}}^{b}=$ {0, 15, 30, 45, −30}°, for the simulation setup with a single elliptic, transversely isotropic, brittle inclusion in a ductile matrix. The contour plots of the crack phase $\phi_{c}$ at fracture, overlaying the solid phases, for the different values of $\psi_{\mathrm{aniso}}^{b}$ are given below and beneath the stress–strain diagram.

**Figure 7:**
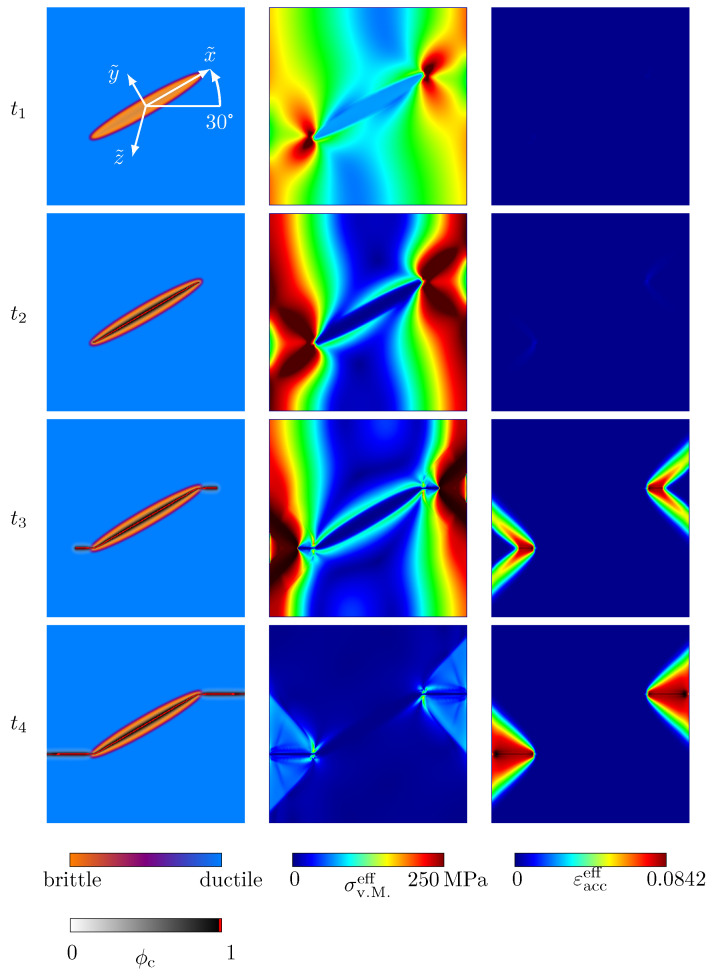
Contour plots of the evolution of the crack phase field $\phi_{c}$ (**first column**) and the resulting von Mises stresses $\sigma_{v.M.}^{\mathrm{eff}}$ (**second column**), together with the effective accumulated plastic strain $\varepsilon_{\mathrm{acc}}^{\mathrm{eff}}$ (**third column**) for the simulation setup with a single elliptic, transversely isotropic, brittle inclusion, embedded in a ductile matrix, for the set of standard parameters, Equation (32). The corresponding macroscopic uniaxial strain states are given in Figure 6a.

First of all, the length parameter to define the width of the diffuse transitions between solid phases is varied for the values *ϵ* = {3, 5, 7}Δ*x*. The influence of the length parameter is investigated in terms of the stress–strain behaviour and the crack morphology (see Figure 6a). In Figure 7, the corresponding contour plots of the equivalent von Mises stress of Equation (23), $\sigma_{v.M.}^{\mathrm{eff}}$, and the effective accumulated plastic strain, $\varepsilon_{\mathrm{acc}}^{\mathrm{eff}}$, for the highlighted states in Figure 6a, as well as the set of standard parameters, are shown. A linear elastic region is present in the stress–strain diagrams, up to a strain of ε≈0.075% ($t_{0}$ − $t_{1}$), before crack nucleation takes place. Based on the evolution of the crack phase, as well as on the stress–strain plots, it can be observed that there are three stages of crack growth. First, a crack spreads within the brittle phase. This crack growth is accompanied by a drop of the stress–strain plot ($t_{1}$–$t_{2}$), which proceeds in a brittle manner. After the stress reaches a critical value ($t_{3}$), the crack tips start to propagate through the diffuse interface. During this stage, the stress–strain plot has an almost constant stress value ($t_{3}$–$t_{4}$). Afterwards, the crack propagates from the upper and lower ends of the ellipsoid and grows horizontally until a complete failure occurs, where the stress–strain plots drop to zero ($t_{4}$–$t_{6}$). After the rupture, residual stresses exist, due to the presence of plastic strains. During crack propagation in the ductile phase, a pronounced nonlinear behaviour can be observed.

The name grey cast iron originates from the fractured surfaces of broken grey cast iron components, which usually have a grey shimmer. Such greyish fracture surfaces are the result of a preferred crack growth along graphite lamellae, due to the significantly lower critical energy release rate of graphite lamellae, compared to the metallic matrix [26]. Under tensile load, graphite lamellae have a negligible loading capacity and therefore act as microcracks even at very small strains [9,28]. The edges of the ruptured graphite lamellae act as stress raisers, which induce local plastic zones [9]. Only when the graphite lamellae have lost their load-bearing capacity does the ductile matrix begin to break. The simulation results show that this behaviour can be mapped by the introduced model. Crack nucleation exclusively takes place within the brittle phase, associated with graphite lamellae, where by the direction of the crack growth along the brittle phase is favoured by the anisotropic formulation of the critical energy release rate, before the crack propagation takes place within the ductile matrix. The crack propagation within the ductile phase is accompanied by local plastic strains occurring in front of the crack tips (see Figure 7) ($t_{3}$). Within the matrix, a Mode-I-type crack growth predominantly occurs in the horizontal direction.

When defining the diffuse width of the solid phase transitions, a slight dependence of the stress–strain curves on the length parameter can be seen, where by a convergence of the material behaviour can be guessed, when the length parameter increases. The crack morphology at fracture is independent of the chosen solid-solid phase transition width, as can be seen by the final crack paths in Figure 6a—bottom. In areas where the simulation domain has multiple coexisting phases, the calculation of the mechanical fields is significantly more expensive, compared to areas with only one phase. Since the length parameter has no strong influence on the simulation results, it is sufficient to choose a length parameter value that is as small as possible but still represents a good compromise between accuracy and calculation time.

Subsequently, the orientation of the brittle ellipsoid is varied. The rotation of the longitudinal axis of the ellipsoid around the *z*-axis of the reference coordinate system for the values $\psi_{\mathrm{aniso}}^{b}=$ {0, 15, 30, 45, −30}° is correlated to the rotation of the $\tilde{x},\tilde{y},\tilde{z}$-coordinate system of the anisotropy formulation. In Figure 6b, the corresponding stress–strain curves and crack paths at the fracture are shown. As one can see, the stress–strain curves are heavily dependent on the orientation of the inclusion. The simulation with a rotation angle of $\psi_{\mathrm{aniso}}^{b}=0{}^{\circ}$ shows the smallest maximum stress value. In analogy to lamellar graphite, the loading takes place exactly orthogonally to the basal planes, so that the highest value of the elastic strain energy coincides with the direction of the lowest crack resistance. The gap between the maximum elastic strain energy and the minimum crack resistance grows as the rotation angle increases, which results in the fact that greater stresses are required to cause cracking. For this reason, the simulation with a rotation angle of $\psi_{\mathrm{aniso}}^{b}=45{}^{\circ}$ has the highest maximum stress. Regardless of the rotation angle, the crack always develops along the longitudinal axes of the brittle particles. When comparing $\psi_{\mathrm{aniso}}^{b}=30{}^{\circ}$ and $\psi_{\mathrm{aniso}}^{b}=-30{}^{\circ}$ in Figure 6b, it can be observed that the resulting stress–strain diagrams for a change of sign of the rotation angle are matching.

**Figure 8:**
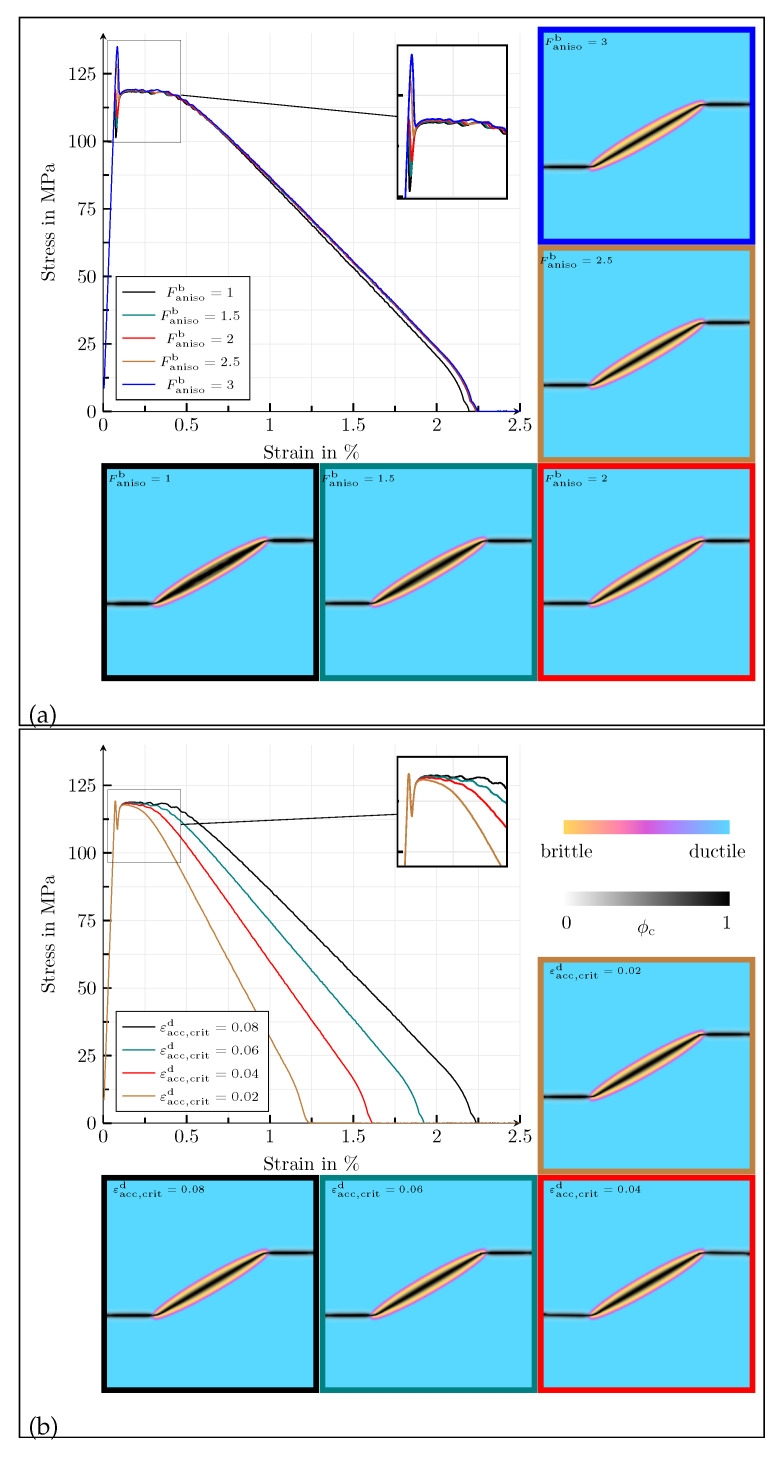
(**a**) Homogenised macroscopic stress–strain responses in the case of uniaxial tension for different values of $F_{\mathrm{aniso}}^{b}=$ {1, 1.5, 2, 2.5, 3}, for the simulation setup with a single elliptic, transversely isotropic, brittle inclusion in a ductile matrix. The contour plots of the crack phase $\phi_{c}$ at fracture, overlaying the solid phases, for the different values of $F_{\mathrm{aniso}}^{b}$ are given below and beneath the stress–strain diagram. (**b**) Homogenised macroscopic stress–strain responses in the case of uniaxial tension for different values of $\varepsilon_{\mathrm{acc},\mathrm{crit}}^{d}=$ {0.08, 0.06, 0.04, 0.02}, for the simulation setup with a single elliptic, transversely isotropic, brittle inclusion in a ductile matrix. The contour plots of the crack phase $\phi_{c}$ at fracture, overlaying the solid phases, for the different values of $\varepsilon_{\mathrm{acc},\mathrm{crit}}^{d}$ are given below and beneath the stress–strain diagram.

The influence of the anisotropy factor on the mechanical behaviour is shown in Figure 8a. The results correspond to the values $F_{\mathrm{aniso}}^{b}=$ {1, 1.5, 2, 2.5, 3}. The stress–strain curves are almost identical. As in the previous example, the increase in the stiffness components of the brittle phase, which is due to the anisotropy factor, only has a small influence on the macroscopic material behaviour, as a microcrack already develops inside the ellipsoid at very small strains. The only difference is observed between the maximum values of the stresses. In the nonlinear area of the diagram, which is characterised by plastic deformations, the graphs are identical. On the scale of the crack phase field, it is noticeable that the crack becomes thinner as the anisotropy factor increases, since the strength of the directional dependence increases.

Finally, the influence of the threshold value $\varepsilon_{\mathrm{acc},\mathrm{crit}}^{d}$ of the ductile phase is investigated for the values $\varepsilon_{\mathrm{acc},\mathrm{crit}}^{d}=$ {0.8, 0.6, 0.4, 0.2}. The corresponding results are given in Figure 8b. Again, a change of the threshold value has no influence on the material behaviour, as long as the crack propagates within the brittle phase, neither with regard to the stress–strain behaviour nor with regard to the crack morphology. In the ductile phase, the crack morphology is also not changed significantly by the threshold value. With an increasing plastic threshold, the elongation at the break rises, as the ductile degradation function depends on $\varepsilon_{\mathrm{acc},\mathrm{crit}}^{d}$, which means that higher plastic deformations are required to cause crack propagation in the ductile phase when $\varepsilon_{\mathrm{acc},\mathrm{crit}}^{d}$ does increase.

### 4.3. Multiphase Simulations in an Idealised Grey Cast Iron Microstructure

**Figure 9:**
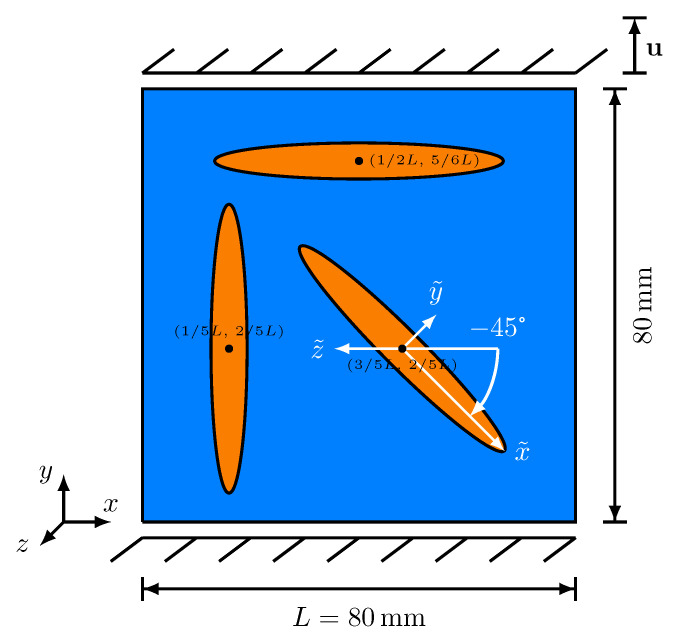
Simulation domain with three differently oriented elliptic, transversely isotropic, brittle inclusions in a ductile matrix, accompanied by the applied mechanical boundary conditions. The anisotropic brittle phases are represented in orange, while the ductile matrix is represented in blue.

As a more realistic application example, the developed model is applied to an idealised grey cast iron microstructure. As simplified representations of graphite lamellae, the simulation domain contains three differently oriented elliptic, transversely isotropic, brittle inclusions in a ductile matrix. The length of 26.67 mm and the width of 3.33 mm are the same for all inclusions. Figure 9 shows the simulation setup and the applied mechanical boundary conditions (see Section 4), which have already been applied in the previous validation cases. Between the solid phases, a diffuse interface, corresponding to a width of ϵ=3Δx, is generated, while no solid-solid phase transformation takes place during the simulations. Since the simulation domain is not a representative volume element in terms of a realistic cast iron with the lamellar graphite microstructure, the brittle particles extend almost through the entire domain.

The material parameters for the ductile matrix and the brittle inclusions are given in Table 3. The anisotropy factor $F_{\mathrm{aniso}}^{b}=1.5$ and the plastic threshold value $\varepsilon_{\mathrm{acc},\mathrm{crit}}^{d}=0.08$ were selected as model parameters. With the anisotropy factor, the unrotated transversal isotropic stiffness tensor of the anisotropic ellipsoids is obtained according to Equation (30).

**Figure 10:**
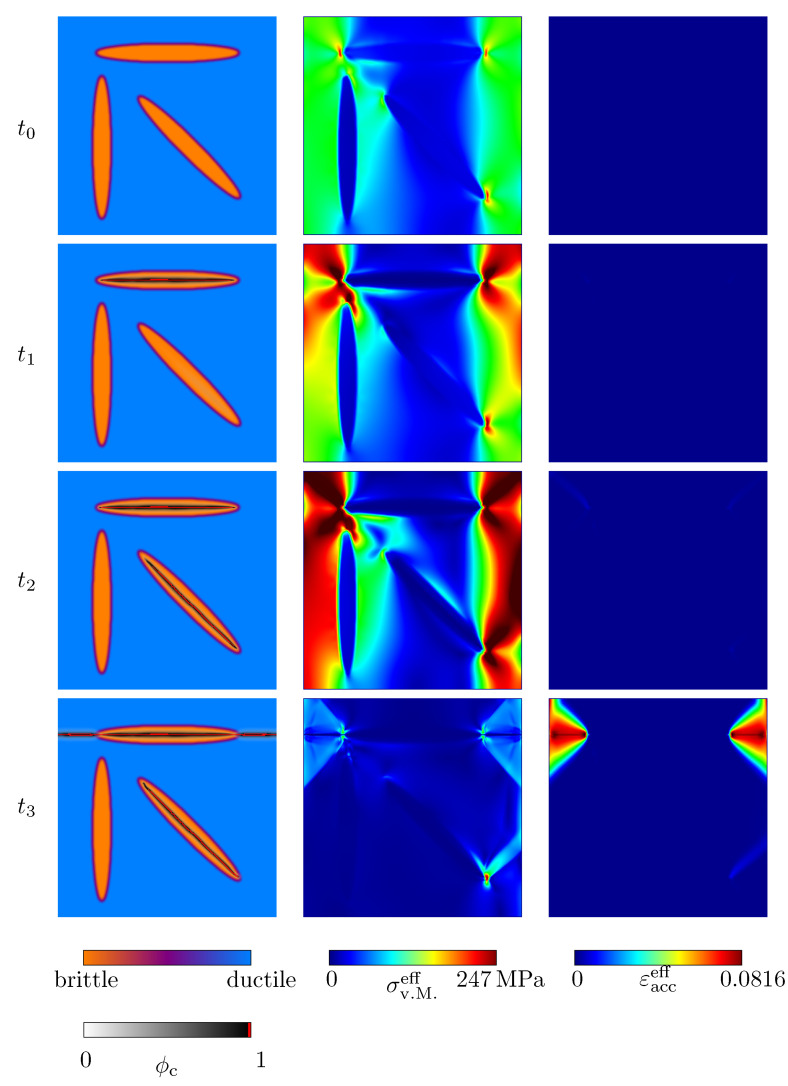
Evolution of the crack phase field $\phi_{c}$ (**first column**) and the resulting von Mises stresses $\sigma_{v.M.}^{\mathrm{eff}}$ (**second column**), together with the effective accumulated plastic strain $\varepsilon_{\mathrm{acc}}^{\mathrm{eff}}$ (**third column**) of an idealised grey cast iron microstructure. Uniaxial tensile load was applied. The corresponding macroscopic uniaxial strain states are given in Figure 12.

**Figure 11:**
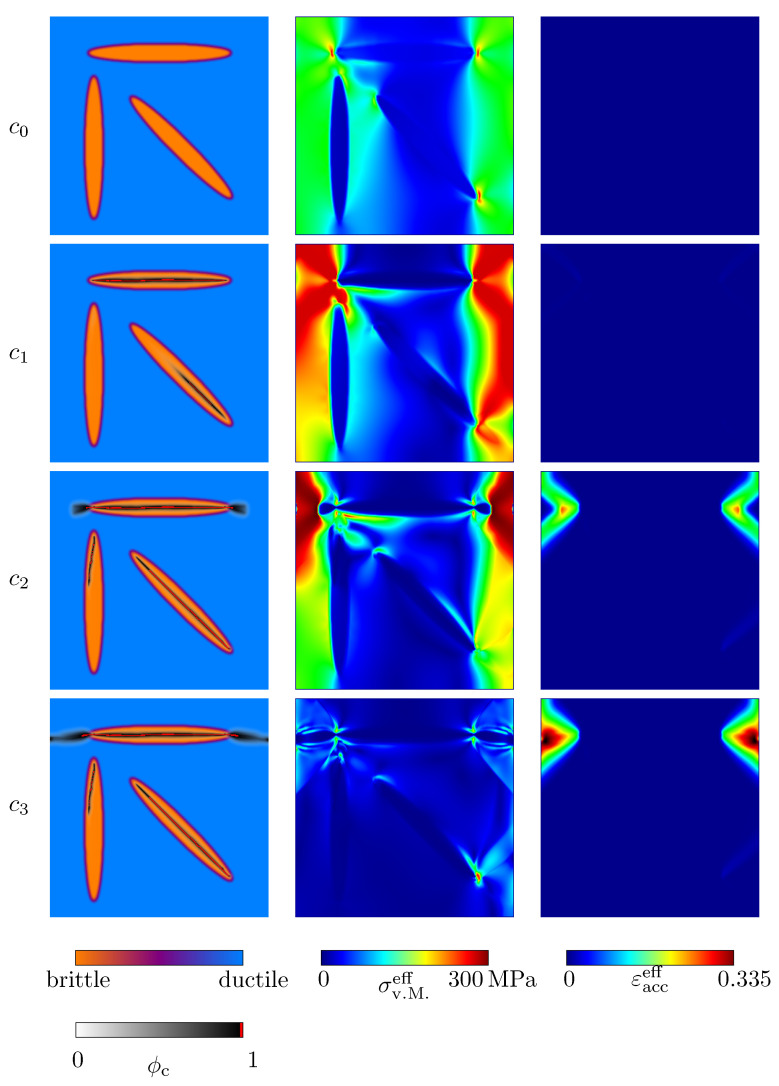
Evolution of the crack phase field $\phi_{c}$ (**first column**) and the resulting von Mises stresses $\sigma_{v.M.}^{\mathrm{eff}}$ (**second column**), together with the effective accumulated plastic strain $\varepsilon_{\mathrm{acc}}^{\mathrm{eff}}$ (**third column**) of an idealised grey cast iron microstructure. Uniaxial compressive load was applied. The corresponding macroscopic uniaxial strain states are given in Figure 12.

**Figure 12:**
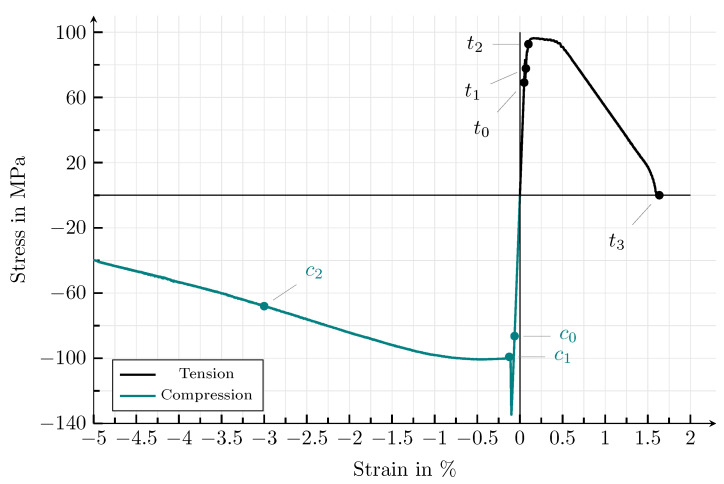
Homogenised macroscopic stress–strain response of a simulation domain, inspired by an idealised cast iron microstructure with lamellar graphite. The diagrams show the stress and strain in the case of uniaxial tension and compression. The dots in the stress–strain diagram correspond to the strain states of the contour plots of Figure 10 and Figure 11. Even though the simulation domain is not a representative volume element, a clear tension–compression dependency of the stress–strain plot can be observed when looking at the peak stresses and the elongations at failure.

Figure 10 and Figure 11 show the evolution of the crack phase field under uniaxial tensile and compressive load. In addition, contour plots of the von Mises stresses $\sigma_{v.M.}^{\mathrm{eff}}$ and the effective accumulated plastic strain, $\varepsilon_{\mathrm{acc}}^{\mathrm{eff}}$, are given. All corresponding states of Figure 10 and Figure 11 are highlighted in the stress–strain plot in Figure 12. In both loading cases, the crack nucleation takes place in the ellipsoid, whose longitudinal axis is oriented orthogonally to the loading direction. The weakest critical energy release rate lies exactly along that longitudinal axis. In analogy to physical reality, in this case, the weak van der Waals bonds of this graphite lamella are mainly stressed, which results in the observed growth behaviour. The crack growth within the horizontal ellipsoid is followed by local stress peaks at the ellipsoid’s edges and associated with plastic deformations of the pearlitic matrix. The described behaviour is consistent with experimental observations [9] and has already been observed in Section 4.2. After the first ellipsoid is fractured, the ellipsoid which shows a rotation of the longitudinal axis by ψ=−45° with respect to the reference coordinate system starts to break. Under compressive load, a crack also develops within the vertical ellipsoid. When pressure is applied, significantly higher plastic deformations occur, as expected. After the specimens are broken, the zones under plastic strain induce a complex distribution of residual stresses, which is typical for grey cast iron [9]. Under tensile load, the crack morphology within the ductile matrix is narrower than under compressive load. Overall, the behaviour observed in the simulations—that microcracks first form within the graphite particles and that these microcracks subsequently coalesce into macrocracks—is consistent with experimental results (see, e.g., [43,44,45]).

With regard to the type of load, important characteristics of the complicated constitutive behaviour of grey cast iron can be found in the simulation results. This includes the nonexistence of a linear elastic regime under tensile load and the tension–compression–stress asymmetry [46]. Figure 12 clearly depicts the asymmetric behaviour under tensile and compressive load, which is typical for cast iron with lamellar graphite on the macroscopic scale [47,48]. Under tensile load, the tested material possesses a brittle behaviour: at an elongation of ε≈0.2% and a stress peak of σ≈95 MPa, crack nucleation takes place. Crack growth occurs without the occurrence of large macroscopic plastic deformations and leads to a fracture at an elongation of ε≈ 1.63%. Keeping the size of the simulation area in mind, these results are in relatively good agreement with the experimental findings documented in the literature. According to Noguchi and Shimizu [49], grey cast iron does not show a pronounced yield strength under tensile load and breaks at a strain between ε = 0.5–1.0%, without visible necking. In grey cast irons, tensile fracture stresses are in the range of σ=100–500 MPa [50]. Under compressive load, a drop in the stress–strain diagram occurs after a strain of ε≈0.2% and a stress peak of σ≈134.5 MPa. Before this point, the course of the curve is linear. Afterwards, a large segment occurs, which shows nonlinear material behaviour, caused by the plastic deformation of the ductile matrix. The elongation at fracture under compressive load is ε>5% and can therefore no longer be properly represented when small deformations are assumed. This different behaviour under tensile and compressive load is mainly driven by the tension–compression split of the elastic strain energy. Under compressive load, the elastic strain energy is considerably smaller compared to the tensile example; therefore, failure of the ductile matrix occurs at significantly higher strains.

## 5. Summary and Outlook

In this work, a small-strain phase-field model capable of predicting crack propagation in systems with anisotropic brittle and ductile constituents was presented. To distinguish between the brittle and ductile crack characteristics, two different formulations of the degradation function are used. As an extension to our previous model formulation [11], the ductile degradation function by Ambati et al. [16] was also introduced. To simulate nonlinear mechanical behaviour, a model, based on the $J_{2}$-plasticity theory is applied, which fulfils the mechanical jump conditions for the phase with the largest volume fraction. Idealised simulation setups were used to investigate the influences of the relevant model parameters on cracks passing through a planar interface between a brittle and a ductile solid phase and for crack development in a domain of a single brittle ellipsoid, embedded in a ductile matrix. Afterwards, uniaxial tension and compression tests were performed in a domain, inspired by a grey cast iron microstructure. It was shown that important mechanical grey cast iron properties can be mapped by the model, which makes it suitable for the simulation of that material group. The model captures the initiation of microcracks within graphite lamellae and the subsequent coalescence of these microcracks by crack growth processes within the ductile matrix to form a fracture. On the macroscopic length scale, the tension–compression load asymmetry of the uniaxial stress–strain response is mapped.

An important and necessary improvement of the model is to make it independent of the regularisation parameter, which defines the width of the diffuse transition between the solid phases and the crack. With such a modification of the model, the crack development in cast iron microstructures could be simulated on its true physical length scale in the millimetre range. This could be done in a similar way as published by Wu and Nguyen [37] but with the additional difficulty that a brittle-anisotropic model formulation, combined with a ductile model formulation, would have to be considered, instead of a brittle-isotropic model formulation.

In future work, we intend to investigate cracking processes in grey cast iron materials under realistic thermomechanical load, following braking operations of truck brake discs. Furthermore, we want to investigate the crack formation in grey cast iron that has undergone the martensitic transformation process. In addition, the use of a finite deformation model would be of interest. Furthermore, the model can be applied or extended to other types of material composites, composed of multiple phases with different mechanical properties such as fibre-reinforced polymer structures, fuel cells, or battery materials.

## Figures and Tables

**Table 1:** Governing equations of the phase-field crack model.

**Mechanical equilibrium**	
Static balance of momentum	divσ=0
Static balance of momentum, at a singular surface	$\left(\sigma^{\alpha }-\sigma^{\beta }\right)n^{\alpha \beta }=0$
Hadamard jump condition	$\left(H^{\alpha }-H^{\beta }\right)=a^{\alpha \beta }⊗n^{\alpha \beta }$
**Plasticity**	
Karush–Kuhn–Tucker system	$\left\{ \begin{array}{c} f_{y}^{\alpha }\leq 0\\ {\dot{\gamma }}^{\alpha }\geq 0\\ f_{y}^{\alpha }{\dot{\gamma }}^{\alpha }=0 \end{array}\right.$
Flow rule	${\dot{\varepsilon }}_{p}^{\alpha }={\dot{\gamma }}^{\alpha }\frac{\partial f_{y}^{\alpha }}{\partial s^{\alpha }}$
Yield function	$f_{y}^{\alpha }=\sqrt{\frac{3}{2}}\left|s^{\alpha }\right|-\left(\sigma_{y,0}^{\alpha }+H^{\alpha }\varepsilon_{\mathrm{acc}}^{\alpha }\right)$
**Crack phase field evolution**	
Karush–Kuhn–Tucker system	$\left\{ \begin{array}{c} g_{c}\geq 0\\ \dot{\phi_{c}}\geq 0\\ g_{c}\dot{\phi_{c}}=0 \end{array}\right.$
Flow rule	$\dot{\phi_{c}}=-Mg_{c}$
Yield function	$g_{c}=\frac{3}{8}(\bar{G_{c}}(\frac{1}{\bar{l}}-2\bar{l}\nabla \;\cdot \;\nabla \phi_{c})$
	$-\nabla \;\cdot \;\frac{\partial \bar{G_{c}}}{\partial \nabla \phi_{c}}(\frac{\phi_{c}}{\bar{l}}+\bar{l}{\left|\nabla \phi_{c}\right|}^{2}))$
	$+\frac{\partial f_{e}}{\partial \phi_{c}}$
**Solid phase evolution**	
Flow rule	$\dot{\phi^{\alpha }}=-h_{s}^{\alpha }\dot{\phi_{c}}$
**Volume fraction conditions of multiphase field approach**	
Sum condition of multiphase-field model	$h_{s}^{\alpha }=\frac{\phi^{\alpha }}{\sum_{\beta =1}^{N}\phi^{\beta }}$
Sum condition of solid phases	$\sum_{\alpha =1}^{N}h_{s}^{\alpha }=1$

**Table 2:** Summary of the most important symbols, categorically grouped.

Symbol	Meaning
$\phi^{\alpha }$	Solid phase fields
$\hat{\phi }$	Phase field-*N*-tuple
$h_{s}^{\alpha }$	Interpolation function for solid phases
F	Free energy functional
*M*	Mobility kinetic coefficient
$\phi_{c}$	Crack phase field
$h_{c}^{\alpha }$	Crack degradation function
$l^{\alpha }$	Phase-inherent regularisation parameter
$\bar{l}$	Interpolated regularisation parameter
$\hat{l}$	Collection of phase-inherent regularisation parameters
$G_{c}^{\alpha }$	Phase-inherent critical energy release rate
$\bar{G_{c}}$	Interpolated critical energy release rate
$G_{c,0}^{\alpha }$	Phase-inherent referential critical energy release rate
$F_{\mathrm{aniso}}^{\alpha }$	Phase-inherent anisotropy factor
$\varepsilon_{\mathrm{acc},\mathrm{crit}}$	Plastic threshold value
$H^{\alpha }$	Phase-inherent displacement gradients
$\hat{H}$	Collection of phase-inherent displacement gradients
$H^{\mathrm{eff}}$	Effective displacement gradient
ε	Total strain tensor
$\varepsilon_{e}$	Elastic strain tensor
$\varepsilon_{e}^{\alpha }$	Phase-inherent elastic strain tensor
$\hat{\varepsilon_{e}}$	Collection of phase-inherent elastic strains tensors
$f_{e}$	Effective elastic strain energy
σ	Stress tensor
$\sigma^{\alpha }$	Phase-inherent stress tensor
$\hat{\sigma }$	Collection of phase-inherent stress tensors
$\sigma^{\mathrm{eff}}$	Effective stress tensor
$s^{\alpha }$	Phase-inherent deviatoric stress tensor
$\varepsilon_{p}$	Plastic strain tensor
$\varepsilon_{p}^{\alpha }$	Phase-inherent plastic strain tensors
$\varepsilon_{\mathrm{acc}}^{\alpha }$	Phase-inherent accumulated plastic strain
$\varepsilon_{\mathrm{acc}}^{\mathrm{eff}}$	Effective accumulated plastic strain
$f_{y}^{\alpha }$	Phase-inherent yield function
$\sigma_{y,0}^{\alpha }$	Phase-inherent yield strength
$H^{\alpha }$	Phase-inherent linear hardening parameter
$f_{p}^{\alpha }$	Phase-inherent plastic energy
$f_{p}$	Effective plastic energy
$n^{\alpha \beta }$	Unit normal vectors between the two phases
a	Rank-1 tensor
$Q_{\mathrm{aniso}}^{\alpha }$	Transformation matrices

**Table 3:** Mechanical parameters for pearlite and graphite, used in the simulations.

Properties	Pearlite	Graphite
Young’s modulus *E*	210.0 GPa	12.40 GPa
Poisson’s ratio ν	0.3	0.1
Yield stress $\sigma_{y,0}$	230.0 MPa	
Hardening modulus *H*	2.1 GPa	
Fracture toughness $K_{\mathrm{Ic}}$	50.0 MPa$\sqrt{m}m$	1.34 MPa$\sqrt{m}m$
Tensile strength $\sigma_{m}$	738.0 MPa	27.6 MPa

## Data Availability

The data presented in this study are available on request from the corresponding author.
